# Exploiting an Epigenetic Resistance Mechanism to PI3 Kinase Inhibition in Leukemic Stem Cells

**DOI:** 10.1101/2025.07.11.663968

**Published:** 2025-07-15

**Authors:** Shira G. Glushakow-Smith, Imit Kaur, Simone Sidoli, Shayda Hemmati, Ellen Angeles, Taneisha Sinclair, Samarpana Chakraborty, Aaliyah Battle, Kristina Ames, Swathi-Rao Narayanagari, Rotila Hyka, Mark Soto, Melissa Tracy, Jayaram Vankudoth, Seiya Kitamura, Linde Miles, Ulrich Steidl, Aditi Shastri, Amit Verma, Kira Gritsman

**Affiliations:** 1 Department of Cell Biology, Albert Einstein College of Medicine, Bronx, NY; 2 Department of Medicine, Montefiore Hospital, Bronx, NY; 3 Department of Oncology, Montefiore Hospital, Bronx, NY; 4 Ruth L. and David S. Gottesman Institute for Stem Cell Research and Regenerative Medicine, Albert Einstein College of Medicine, Bronx, NY; 5 Department of Biochemistry, Albert Einstein College of Medicine, Bronx, NY; 6 Department of Developmental and Molecular Biology, Albert Einstein College of Medicine, Bronx, NY; 7 Division of Experimental Hematology and Cancer Biology, Cincinnati Children’s Hospital Medical Center, Cincinnati OH; 8 Department of Pediatrics, University of Cincinnati, Cincinnati, OH; 9 Center for Tumor Dormancy, Albert Einstein College of Medicine, Bronx, NY

**Keywords:** acute myeloid leukemia, PI3K, EZH2, PRC2

## Abstract

Acquired non-genetic resistance mechanisms to existing therapies contribute to poor outcomes for acute myeloid leukemia (AML) patients, and inability to target leukemic stem cells (LSCs) can lead to relapse. To overcome these challenges, we tested whether LSCs have dependencies on PI3 kinase (PI3K). We found that LSCs are susceptible to isoform-selective targeting of PI3K and are particularly dependent on the P110 alpha isoform of PI3K. We discovered that PI3K inactivation leads to dynamic changes in EZH2/PRC2 function in leukemic cells, and we uncovered downregulation of EZH2 protein levels as a resistance mechanism in response to PI3K inhibition. We found that PI3K inhibition in AML cells can lead to compensatory upregulation of EZH1, and that EZH1 knockdown can sensitize AML cells to PI3K inhibition. We leveraged this resistance mechanism by combining a PI3K inhibitor with an EZH1/2 dual inhibitor, which successfully overcomes the acquired resistance and leads to sustained targeting of AML cells ex vivo and in murine AML and PDX models in vivo. This study identifies a promising novel therapeutic regimen for targeting LSCs in AML.

## Introduction

Acute Myeloid Leukemia (AML) is a devastating disease for which the prognosis has not improved significantly over the last 10 years. This is due to the high toxicity of currently available treatments and the fact that 50–70% of AML patients will relapse after achieving complete remission [1]. One reason for this is that most current therapeutic approaches for AML are focused on targeting the highly proliferative leukemic blasts, which make up the bulk of the disease. However, this often leaves behind the leukemic stem cells (LSCs) that can contribute to minimal residual disease (MRD), which is associated with relapse and poor prognosis in AML [2]. Furthermore, both LSCs and blasts often develop resistance to standard AML treatments. Resistance to cancer therapeutics may arise from genetic mechanisms, such as selective pressure for specific mutations. However, a less well understood mechanism of resistance to therapy are non-genetic mechanisms, which include epigenetic changes or transcriptional plasticity as a result of therapeutic pressure[3].

Additionally, AML is molecularly heterogeneous, with a variety of cytogenetic alterations and driver mutations[4]. While targeted therapeutics for some of these mutations are available, this heterogeneity makes it difficult to develop more universal therapeutics for AML, and emergence of resistant clones with different mutations is common. One of the two most common classes of driver mutations is in activated signaling molecules, such as FLT3, C-KIT, or RAS. Activation of the PI3K/AKT pathway, which is downstream of all of these signaling molecules, is observed in up to 80% of AML cases[5], making this pathway an attractive therapeutic target for more universal therapeutics. PI3K and AKT are rarely themselves mutated in AML, but AKT is frequently phosphorylated, including in LSCs [6–8]. Additionally, we and others have shown that activation of AKT in mouse hematopoietic cells can drive both AML and T-ALL [9–11].

The second most common class of driver mutations in AML is in epigenetic regulators, such as DNA methyltransferases and histone methyltransferases [4], which are observed in over 75% of AMLs. Among epigenetic regulators implicated in AML, the histone methyltransferase Enhancer of Zeste Homolog 2 (EZH2) has been widely studied not only in AML, but also in a variety of other cancers. EZH2 is a core component of the Polycomb Repressive Complex 2 (PRC2), which is responsible for the deposition of the repressive H3K27me3 mark. EZH2 can be directly mutated or it can be impacted by other mutations that either disrupt or enhance its function[12, 13], and it can function as either a tumor suppressor or oncogene, depending on the context[13].

Class I PI3Ks are a family of heterodimeric lipid kinases composed of a regulatory subunit and a catalytic subunit [14]. These kinases are responsible for directly activating signal transduction pathways via recruitment to the plasma membrane by receptor tyrosine kinases, as well as other phosphorylated proteins. This begins a signaling cascade in which phosphatidyl inositol phosphate-2 (PIP2) is converted to PIP3, and the subsequent accumulation of PIP3 allows for the signaling transmission into the cell and activation of downstream effectors such as AKT, a serine/threonine kinase which regulates many key processes in the cell [14, 15]. PI3K/AKT pathway inhibition has been demonstrated to inhibit the proliferation of AML cells in preclinical studies [16–19]. However, despite multiple early phase clinical trials [20], no PI3K or AKT inhibitors have been approved for AML treatment to date. This could be due to the inability to achieve sufficient potency in AML cells without excessive toxicity, as the PI3K/AKT pathway is also required for many normal processes in healthy cells. Furthermore, therapeutic resistance could have been an issue, and potential resistance mechanisms to PI3K inhibition in AML are poorly understood.

In the hematopoietic compartment, PI3K signaling is important for maintaining HSC homeostasis [9–11, 21], so it is important to determine a safe way to target this pathway in leukemia patients. Of note, there are three catalytic subunits of Class 1A PI3K (P110α, β, and δ), encoded by *Pik3ca*, *Pik3cb*, and *Pik3cd*, respectively. These isoforms are biochemically interchangeable, as they bind to the same regulatory subunits p85α and p85β. Recent findings have shown leukemic dependency of Class 1B PI3K (P110γ) in certain acute leukemias [22–24]. In other forms of cancer, isoform-selective inhibition of PI3K has been shown to be an effective therapeutic strategy, and several isoform-selective inhibitors have been approved by the FDA for use in patients with chronic lymphocytic leukemia, lymphoma, or breast cancer [25].

To determine if isoform-selective inhibition could also be a safe and effective strategy in AML, we previously generated conditional knockout models for individual PI3K isoforms in mouse hematopoietic cells and in leukemic cells [26, 27]. We found that myeloid leukemias can have isoform-selective dependencies on PI3K [26, 27]. In order to determine a safe way to target the PI3K pathway in LSCs, we asked whether LSCs in AML could also have isoform-selective PI3K dependencies.

Here we report that LSCs are selectively dependent on the P110α isoform of PI3K, and that PI3K inactivation, either genetically or pharmacologically, promotes myeloid differentiation and loss of self-renewal. Our studies also uncovered a non-genetic mechanism of resistance to PI3K inactivation - downregulation of EZH2 protein levels, which makes resistant cells more dependent upon another methyltransferase EZH1. We found that the combination of a PI3K inhibitor with an EZH1/2 inhibitor can effectively leverage this acquired resistance mechanism to deplete the LSC pool in AML.

## Results

### PI3K inactivation impairs leukemic stem cell self-renewal and promotes myeloid differentiation

**Figure 1.**
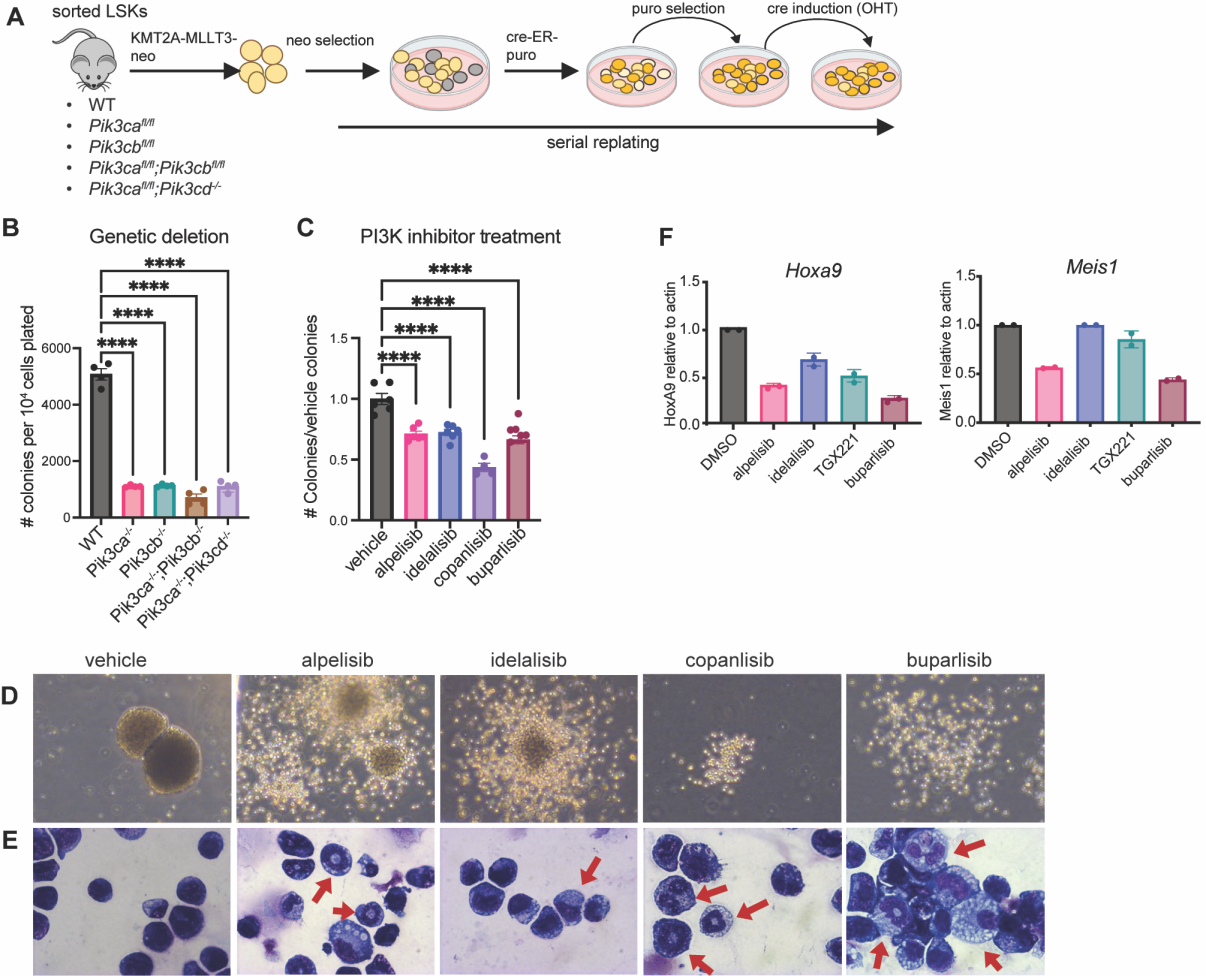
PI3K Disruption Impairs Leukemic Stem Cell Self-Renewal and Promotes Myeloid Differentiation **(A)** Experimental schematic for generation of PI3K KO LSC colonies with inducible excision of PI3K isoforms **(B)** KMT2A-MLLT3 leukemic stem cell colonies after 4 weeks of serial re-plating at one week after acute excision of PI3K isoforms (n=4) **(C-F)** KMT2A-MLLT3 leukemic stem cell colonies in the presence of PI3K inhibitors (alpelisib = a selective, idelalisib = d selective, copanlisib = a/d selective, TGX221 = b selective, buparlisib = pan-PI3K inhibitor) **(C)** Colony counts normalized to vehicle control. **(D)** Representative colony images captured using 400x objective (Top) Representative images of cytospins made from colonies captured using 630x objective (Bottom). Red arrows indicate cells with monocytic or neutrophilic morphologies **(E)** qPCR on cells from colonies (n=2). Each experiment was performed at least three times with similar results. Each value is presented as mean +/− standard error of the mean (SEM). One-way ANOVA test with Tukey’s multiple comparisons was used in B and C. ****P ≤ 0.0001

To study the effects of targeting PI3K in leukemic stem cells (LSCs), we performed colony forming assays on sorted Lin-Sca1+c-Kit+ (LSK) cells from *Pik3ca* ^lox/lox^, *Pik3cb*^lox/lox^, *Pik3ca*^lox/lox^;*Pik3cb*^lox/lox^, or *Pik3ca* ^lox/lox^;*Pik3cd*^−/−^ mice transduced with either the KMT2A-MLLT3-NEO (MLL-AF9-NEO) or the AML1-ETO9A-GFP retrovirus (Figure 1, Supplementary Figure 1).

We acutely excised the PI3K alleles by introducing a Cre-ER-puro retrovirus after 4 weeks of serial replating, selection with puromycin, and induction with 4-orthohydroxytamoxifen (4-OHT) (Figure 1A). We found that deletion of either *Pik3ca*, *Pik3cb*, or pairs of isoforms from leukemic stem cells caused a significant reduction in the serial replating capacity of both KMT2A-MLLT3 and AML1-ETO9a cells (Figure 1B, Supplementary Figure 1, A and B). However, while transduction of P110δ knockout (*Pik3cd*^−/−^) LSK cells with KMT2A-MLLT3 initially generated fewer colonies than WT cells, *Pik3cd* deletion alone did not abrogate serial replating (Supplementary Figure 1A).

We next examined the effects of pharmacologic inhibition of PI3K on LSCs by performing serial replating assays on WT LSK cells transduced with the KMT2A-MLLT3-Neo retrovirus in the presence of either isoform-selective PI3K inhibitors (P110α: alpelisib[28], P110β: TGX221[29], P110δ:idelalisib[30], P110α/δ: copanlisib[31]), P110γ: eganelisib[22] or the pan-PI3K inhibitor buparlisib[32] (Supplementary Figure 1C). Here we observed that pharmacologic inhibition of individual PI3K isoforms was sufficient to significantly impair self-renewal of KMT2A-MLLT3 cells (Figure 1C, Supplementary Figure 1D). We also obtained similar results with PI3K inhibitor treatment of AML1-ETO9A-puro transduced LSCs in serial replating assays (Supplementary Figure 1, C and E). Interestingly, the morphology of KMT2A-MLLT3 colonies indicated signs of myeloid differentiation upon treatment with PI3K inhibitors, which was corroborated by morphologic analysis of individual cells from the colonies on cytospins, showing some features of monocytes or neutrophils (Figure 1, D and E). Additionally, quantitative RT-PCR (qPCR) on LSC colonies at seven days after treatment with alpelisib or buparlisib revealed a decrease in the expression of HoxA9 and Meis1, which have been implicated as self-renewal genes in the pathogenesis of KMT2A-MLLT3 AML [33] (Figure 1F). Consistent with these findings in mouse LSCs, the PI3K inhibitor copanlisib also induced myeloid differentiation in the human AML cell lines MOLM14, NOMO1 and OCI-AML3, as evidenced by changes in morphology and increased expression of the monocytic markers CD15 and CD11b (Supplementary Figure 2).

### PI3K deletion depletes functional leukemic stem cells in vivo

**Figure 2:**
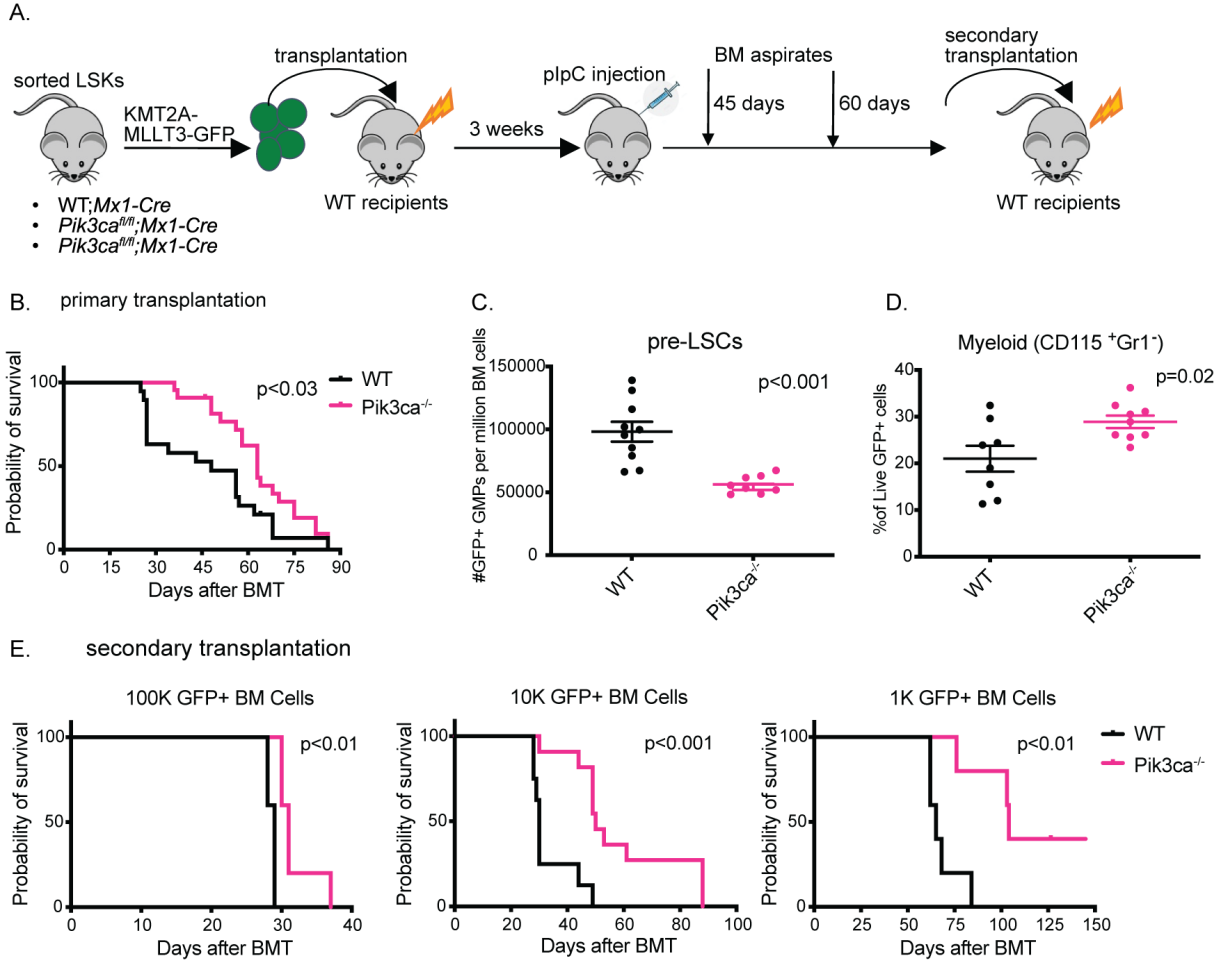
PI3K Deletion Impairs Leukemic Stem Cell Function in vivo **(A)** Experimental schematic for KMT2A-MLLT3 retroviral transduction and transplantation assay with acute excision of PI3K isoforms **(B)** Kaplan-Meier survival curve from primary transplantation. Log-rank analysis was used. (n = 19–22 mice per group) **(C)** Flow cytometry analysis of GFP+ GMPs from 45 day bone marrow (BM) aspirates **(D)** Flow cytometry analysis of 60 day BM aspirates gated on GFP+ cells. (n=8–10 per group) Each value is presented as mean +/− standard error of the mean (SEM). Unpaired t-test was used in C and D. **(E)** Kaplan-Meier survival curves from secondary bone marrow transplant (BMT) with limiting numbers of GFP+ leukemic cells transplanted (n= 5–10 mice per group). Log-rank analysis was used. Each experiment was performed at least three times with similar results

We next wanted to analyze the impact of targeting individual PI3K isoforms in leukemic progression in vivo. To do this, we injected sorted LSK cells from *Pik3ca*^lox/lox^;Mx1-Cre or *Pik3cb*^lox/lox^;Mx1-Cre mice transduced with the KMT2A-MLLT3-GFP retrovirus into lethally irradiated C57Bl/6 recipients (Figure 2A). We found that deletion of *Pik3cb* in leukemic mice had no significant effect on survival or disease progression (Supplementary Figure 3A). Additionally, deletion of *Pik3cb* had no effect on leukemia initiating cell (LIC) frequency as tested by secondary transplantation (Supplementary Figure 3B). We also did not observe any significant changes in spleen weight, though liver weights were slightly decreased in *Pik3cb*^−/−^ mice (Supplementary Figure 3C). We confirmed by PCR that the leukemic cells in *Pik3cb*^−/−^ mice did have excision of exon 2 of *Pik3cb* as expected (Supplementary Figure 3D)[34]. This suggests that PI3Kβ does not play an important role in KMT2A-MLLT3 AML in vivo.

In contrast, deletion of *Pik3ca* significantly prolonged survival in the KMT2A-MLLT3 mice compared to WT, though they eventually developed a similar AML phenotype (Figure 2B, Supplementary Figure 4, A-C). To follow disease evolution, we performed bone marrow aspirates on transplanted mice at two pre-clinical stages, before they showed clinical signs of disease. At the earliest pre-clinical time point, *Pik3ca* deletion resulted in a decrease in the number or immunophenotypically defined LSCs (Figure 2C, Supplementary Figure 4D), defined as GFP+ granulocyte-macrophage progenitors for the KMT2A-MLLT3 AML model [33]. At the second pre-clinical time point, we also observed an increase in expression of the monocytic marker CD115 in bone marrow aspirates from the *Pik3ca*^−/−^ group (Figure 2D). This data suggests that *Pik3ca* plays an important role in maintaining LSCs in vivo. To determine whether *Pik3ca* is also required to maintain functional leukemia-initiating cells (LICs), we performed secondary transplantation of decreasing doses of GFP+ leukemic cells from the primary transplant recipients into sub-lethally irradiated mice. We found that at each dose of leukemic cells transplanted, survival was significantly prolonged in the recipients of *Pik3ca*-deleted leukemic cells, consistent with a depletion in functional LICs (Figure 2E).

### PI3K inactivation impairs LSC function via changes in phosphorylation of EZH2 at Ser21

**Figure 3:**
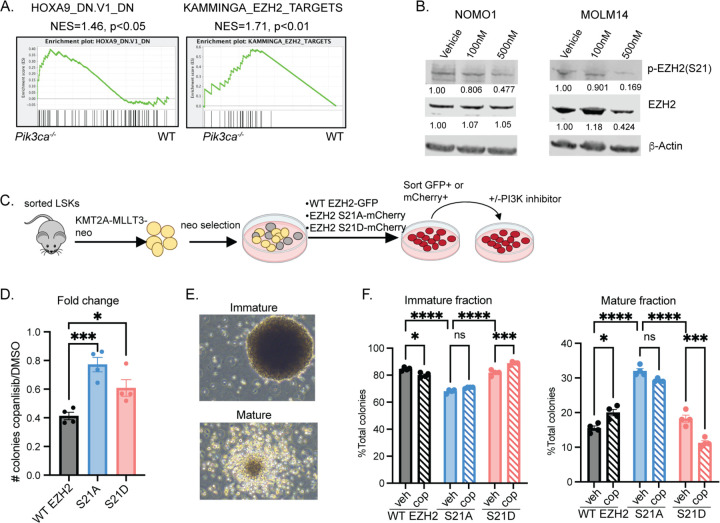
PI3K Inactivation impairs leukemic cell function via changes in phosphorylation of EZH2 at Ser21 **(A)** GSEA plots from sorted GFP+ GMPs from 45-day bone marrow aspirates from primary KMT2A-MLLT3 transplant recipients **(B)** Western blot showing decrease in pEZH2-Ser21 levels after 3 hours of PI3K inhibition with copanlisib at indicated doses in NOMO1 and MOLM14 cells. Quantification of pEZH2 relative to b-actin normalized to DMSO control is reported under each blot **(C)** Experimental schematic for colony assay with overexpression of WT EZH2 EZH2-S21A mutant or EZH2-S21D mutant **(D)** Fold change of colony counts in EZH2 WT or EZH2 mutant colonies treated with copanlisib compared to untreated (n= 4 per group) **(E)** Representative images of KMT2A-MLLT3 leukemic stem cell colonies expressing WT or S21 mutant EZH in the presence of copanlisib, captured with 400x objective **(F)** Percentage of immature and mature colonies (n= 4 per group). Each experiment was performed at least twice with similar results. Each value is presented as mean +/− standard error of the mean (SEM). One-way ANOVA test with Tukey’s multiple comparisons was used for D and F. ****P ≤ 0.0001 ***P ≤ 0.001 *P ≤0.05

To better understand the mechanism through which *Pik3ca* deletion leads to a reduction in functional LSCs, we sorted GFP+ GMPs from preclinical bone marrow aspirates of KMT2A-MLLT3-GFP primary transplant mice (Supplementary Figure 4D) and performed DNA microarray analysis in triplicate using the Affymetrix Mouse Transcriptome Assay 1.0 kit. Gene Set Enrichment Analysis (GSEA)[35, 36] of *Pik3ca*^−/−^ pre-LSCs compared with WT pre-LSCs revealed downregulation of several AKT and AKT-MTOR gene signatures, as expected (Supplementary Figure 5A). This GSEA also corroborated our colony qPCR data, with significant enrichment of a HOXA9 knockdown signature (HOXA9_DN.V1_DN) in *Pik3ca*^−/−^ pre-LSCs, consistent with loss of self-renewal (Supplementary Figure 5B, Figure 3A). Interestingly, we also observed enrichment of the KAMMINGA_EZH2_TARGETS signature in *Pik3ca*^−/−^ pre-LSCs (Supplementary Figure 5B, Figure 3A). In murine models of KMT2A-MLLT3 AML, inactivation of EZH2 and other components of PRC2 compromises leukemic proliferation and prolongs survival [37, 38]. It is also known that AKT phosphorylates EZH2 at Ser21, causing inhibition of EZH2 activity, leading to loss of the H3K27me3 repressive mark [39].

To determine whether P110α regulates LSC function via EZH2 phosphorylation, we performed Western analysis on KMT2A-MLLT3 human cell lines after treatment with the PI3K inhibitor copanlisib. As expected, copanlisib reduced p-EZH2 levels at Ser21 in both MOLM14 and NOMO1 AML cell lines (Figure 3B). To determine if the effects of PI3K inhibition are dependent on its impact on EZH2 phosphorylation at Ser21, we transduced KMT2A-MLLT3 LSCs with a retrovirus expressing either WT EZH2-GFP, a non-phosphorylatable mutant of EZH2 (EZH2-S21A-mCherry), or a phosphomimetic mutant of EZH2 (EZH2-S21D-mCherry) [39]. We then performed colony assays on cells sorted for GFP (for WT EZH2) or mCherry (for EZH2-S21A or EZH2-S21D) in the presence or absence of copanlisib (Figure 3C). LSCs transduced with WT EZH2 were responsive to PI3K inhibition (Figure 3D, Supplementary Figure 6A). However, LSCs transduced with either EZH2-S21A or EZH2-S21D mutant retrovirus showed a significantly decreased response to PI3K inhibition (Figure 3D, Supplementary Figure 6A), indicating that de-phosphorylation of the Ser21 site of EZH2 is at least one key mechanism for the effects of PI3K inhibition on LSC self-renewal. Morphologic evaluation revealed that S21A mutant colonies appear more mature than WT EZH2 colonies, which was unaffected by PI3K inhibitor treatment (Figure 3, E and F). This suggests that EZH2 dephosphorylation via PI3K inactivation promotes differentiation and loss of LSC self-renewal.

### Dynamic changes in EZH2 regulation contribute to AML resistance to PI3K inhibition

**Figure 4:**
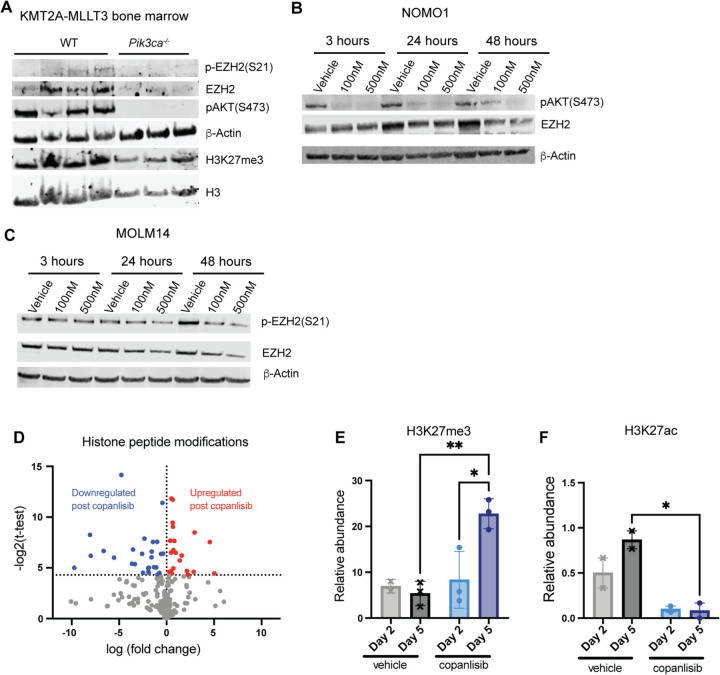
Dynamic Changes in EZH2 Regulation Contribute to AML Resistance to PI3K Inhibition **(A)** Western Blot analysis on bone marrow cells from moribund primary KMT2A-MLLT3 transplant recipient leukemic mice **(B)** Western blot analysis on NOMO1 cells treated for 2 days with 2 doses of copanlisib. Quantification of EZH2 levels relative to b-Actin normalized to DMSO control reported under the EZH2 bands **(C)** Western blot analysis on MOLM14 cells treated for 2 days with 2 doses of copanlisib. Quantification of EZH2 levels relative to b-Actin normalized to DMSO control. Experiment performed at least three times with similar results. **(D)** Volcano plot highlighting the changes in histone peptide modifications in NOMO1 cells after 5 days of treatment with 500nM copanlisib **(E)** Relative abundance of the H3K27me3 mark in NOMO1 cells treated with 500nM copanlisib **(F)** Relative abundance of the H3K27Ac mark in NOMO1 cells treated with 500nM copanlisib. Each value is presented as mean +/− standard error of the mean (SEM). (n = 3 per group) One-way ANOVA test with Tukey’s multiple comparisons was used in E and F. **P ≤ 0.01 *P ≤ 0.05. These experiments were performed two times with similar results.

Although PI3K deletion prolonged survival in the KMT2A-MLLT3 AML model, *Pik3ca* – deleted mice eventually still develop leukemia with a similar phenotype as control WT;Mx1-Cre mice (Figure 2B, Supplementary Figure 4, A-C). To uncover possible resistance mechanisms to targeting PI3K in leukemic cells, we performed Western analysis on bone marrow cells from moribund leukemic primary transplant recipient mice. First, to determine whether other isoforms of PI3K are compensating for loss of *Pik3ca*, we examined AKT phosphorylation at Ser473. Surprisingly, we observed a complete loss of AKT phosphorylation in *Pik3ca*^*−/−*^ leukemic cells (Figure 4A), indicating that compensation by other PI3K isoforms is not a likely mechanism of resistance. Given our transcriptome data showing potential effects of *Pik3ca* deletion on EZH2, we probed for phospho-EZH2(S21) and total EZH2 protein levels. As expected, we were unable to detect any EZH2 phosphorylation in *Pik3ca*^−/−^ leukemic cells (Figure 4A). We were surprised to find that total EZH2 protein levels were also decreased in *Pik3ca*^−/−^ leukemic cells compared to WT, indicating that a loss of EZH2 protein may be a potential resistance mechanism to targeting PI3K (Figure 4A).

To determine if we could recapitulate this dynamic regulation of EZH2 in response to PI3K inactivation in AML cell lines, we treated NOMO1 and MOLM14 cells with copanlisib over the course of two days. Western analysis revealed a decrease in total EZH2 protein levels only after 24 hours of exposure to the PI3K inhibitor, with the greatest decrease observed within 48 hours (Figure 4, B and C, Supplementary Figure 6B). To functionally examine the results of EZH2/PRC2 regulation as a result of PI3K inhibition, we performed histone proteomics on NOMO1 cells after treatment with copanlisib for 2 or 5 days. We found an increasing number of significantly dysregulated histone peptide modifications after prolonged exposure to PI3K inhibitors (Figure 4D). Importantly, we observed a significant increase in H3K27me3 relative abundance and a corresponding decrease in abundance of the H3K27ac activating mark post treatment with copanlisib, indicating an initial restoration of EZH2 and PRC2 canonical function, as would be expected due to de-phosphorylation of EZH2 (Figure 4, E and F).

### PI3K inhibition cooperates with EZH1/2 dual inhibition to target leukemic cells

**Figure 5:**
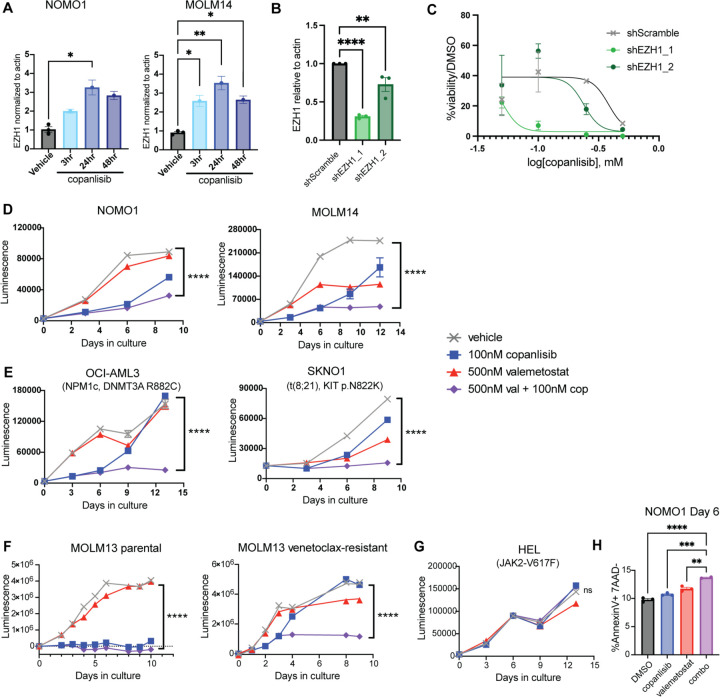
PI3K Inhibitors Cooperate with EZH1/2 Dual Inhibition to Target LSCs **(A)** qPCR testing EZH1 levels in NOMO1 and MOLM14 cells treated with 200nM Copanlisib over the course of 2 days (n = 3) **(B)** Dose response curve on day 11 of treatment of NOMO1 shEZH1 lines with copanlisib treatment. qPCR showing knockdown of EZH1 levels in NOMO1 shEZH1 cells is on the right (n= 3) **(C-F)** Proliferation assays with copanlisib +/− valemetostat treatment of KMT2A-rearranged AML cell lines. Each experiment performed at least three times with similar results. **(C)** and non-KMT2A-rearranged AML cell lines. **(D,F)** and MOLM13 parental and venetoclax resistant AML cell lines **(E) (G)** Representative gating strategy for identifying apoptotic cells in NOMO1 cells after 6 days of treatment (left). Quantification of apoptotic populations in NOMO1 cells after 6 days of treatment (n = 3). Each experiment was performed at least two times with similar results. Each value is presented at mean +/− standard error of the mean (SEM). One-way ANOVA test with Tukey’s multiple comparisons was used in A, B, and F. ****P ≤ 0.0001 ***P ≤ 0.001 **P<0.01 *P ≤ 0.05 ns = not significant.

Previous studies have shown that when EZH2 is compromised, its homologue EZH1 becomes essential for leukemic cell function [40, 41]. To determine if EZH1 might be compensating for loss of EZH2 after PI3K inhibition, we examined the expression of EZH1 in copanlisib-treated AML cell lines by qPCR. We observed a time-dependent upregulation of EZH1 RNA expression in both NOMO1 and MOLM14 cells after copanlisib treatment (Figure 5A). We also confirmed an increase in EZH1 protein expression in MOLM14 cells after 48 hours of copanlisib treatment (Supplementary Figure 7A). To determine if EZH1 can compensate for EZH2 loss in the setting of PI3K inhibition in AML cells, we performed shRNA knockdown of EZH1 in NOMO1 cells. Using two different shRNAs targeting EZH1, we generated stable NOMO1 cells with EZH1 knockdown, as confirmed with qPCR and Western Blot analysis (Figure 5B, Supplementary Figure 7B). Proliferation assays revealed that EZH1 knockdown can sensitize NOMO1 cells to copanlisib (Figure 5C). Importantly, we found that knockdown of EZH1 had no impact on cell proliferation at baseline (Supplementary Figure 7C). However, the shEZH1 cells were more sensitive to copanlisib in a dose dependent manner (Figure 5C, Supplementary Figure 7D).

We hypothesized that pharmacologic inhibition of EZH1 could potentially overcome the resistance to PI3K inhibition that we observed in AML cells driven by the loss of EZH2 and lead to synthetic lethality in PI3K inhibitor-treated cells. To this end, we tested the EZH1/2 dual inhibitor valemetostat, which has been shown to have preclinical activity in AML[37, 42]. We combined valemetostat with copanlisib in Cell Titer Glo proliferation assays in multiple AML cell lines. Our data suggest that in most cases, copanlisib treatment alone was initially able to suppress proliferation, but that within a week of treatment proliferation increased again (Figure 5, D and E). However, when copanlisib was combined with valemetostat, proliferation was suppressed for the duration of the experiment (Figure 5, D and E). Importantly, this was observed in a variety of AML cell lines with diverse molecular and cytogenetic profiles, both KMT2A-rearranged (Figure 5D) and not KMT2A-rearranged (Figure 5E). This suggests that the efficacy of this drug combination is not limited to KMT2A-rearranged AML. Additionally, as venetoclax resistance is becoming a major clinical problem in AML treatment, we also tested our drug combination in the venetoclax-resistant cell line MOLM-13VR. We found that while parental MOLM13 cells are sensitive to copanlisib alone and the combination treatment, MOLM-13VR cells become resistant to copanlisib but are still sensitive to the copanlisib and valemetostat combination treatment (Figure 5F). However, we did observe resistance to this combination treatment in HEL cells, indicating that the anti-proliferative effects of this drug combination are not due to nonspecific cytotoxic effects (Figure 5G).

To determine how this combination treatment impacts proliferation of leukemic cells, we examined myeloid differentiation, cell cycling, and apoptosis by flow cytometry. As our data indicates that PI3K inhibitors alone are able to induce differentiation, we first tested whether the combination treatment promotes more terminal differentiation of leukemic cells. Flow cytometry analysis showed no significant differences in myeloid marker expression with combination drug treatment compared to the PI3K inhibitor alone, so the combination effects of our treatment are unlikely to be a result of differentiation effects (Supplementary Figure 8, A and B). Next, we performed cell cycle analysis using Hoechst 33342 vs Ki-67. After 48 hours of treatment, we observed an increase in the proportion of leukemic cells in G0 cell cycle arrest in our combination treated group compared to either single agent or the vehicle control in both NOMO1 and OCI-AML3 cells (Supplementary Figure 8, C and D). Cytospins of leukemic cells treated for six days showed morphological changes consistent with cell death (Supplementary Figure 8E). This led us to perform apoptosis assays with Annexin V and 7-AAD to determine if our combination treatment is inducing synthetic lethality. We found that after six days of treatment, our combination-treated cells exhibited significantly higher levels of apoptosis in NOMO1 cells compared to either single agent or the vehicle control (Figure 5H, Supplementary Figure 8F). This suggests that the combination of copanlisib and valemetostat initially induces cell cycle arrest in AML cells, ultimately leading to increased apoptosis.

### The PI3K inhibitor copanlisib cooperates with the EZH1/2 dual inhibitor valemetostat to target LSCs in vivo

**Figure 6:**
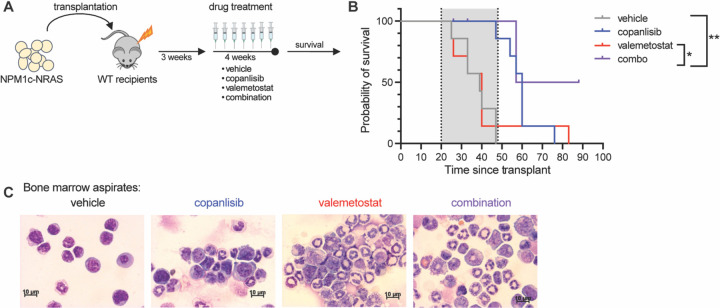
PI3K Inhibition Cooperates with EZH1/2 Dual Inhibition to Target LSCs in NPM1c-NRAS AML in vivo **(A)** Experimental schematic for bone marrow transplantation assay of NPM1c-NRAS AML cells with *in vivo* drug treatment **(B)** Survival curves (n = 6–8 per group). **(C)** Representative cytospin images made from Bone Marrow Aspirates taken at 2 weeks post treatment start. Each value is presented at mean +/− standard error of the mean (SEM). One-way ANOVA test with Tukey’s multiple comparisons was used in D and F. *P ≤ 0.05 ns = not significant.

Given the promising results in our ex vivo experiments, we next sought to confirm these results in vivo. To test this, we injected bone marrow cells from CD45.2 NPM1^LSL-CA/+;^NRAS^LSL-G12D^ leukemic mice[43] into lethally irradiated CD45.1 WT recipients. Once engraftment was confirmed, we randomized the mice by %CD45.2 in the peripheral blood and then treated them for four weeks with 6mg/kg copanlisib intraperitoneally, 100mg/kg valemetostat by oral gavage, or the combination (Figure 6A). The valemetostat used in vivo was synthesized by the Albert Einstein College of Medicine Chemical Synthesis Core Facility and was confirmed to reduce H3K27me3 in MOLM14 cells by Western blot (Supplementary Figure 9A) and to reduce proliferation in MOLM14 cells, alone and in combination with copanlisib (Supplementary Figure 9B). After four weeks of treatment, we continued to monitor for survival any remaining animals and then concluded the experiment at 8 weeks post treatment (Figure 6, A and B). After two weeks of treatment, we performed preclinical bone marrow aspiration for analysis. Flow cytometry analysis of preclinical bone marrow aspirates showed no differences in disease burden, as demonstrated by similar levels of %CD45.2 across the treatment groups (Supplementary Figure 10A). Intracellular flow cytometry indicated that valemetostat is successfully able to achieve on target effects in immunophenotypically defined LSCs, resulting in a decrease in H3K27me3 levels in these cells (Supplementary Figure 10, B and C). Cytospins made from the bone marrow aspirates showed significant morphological changes in cells treated with valemetostat alone and in combination with copanlisib consistent with myeloid differentiation (Figure 6C). Upon completion of the experiment at 8 weeks post treatment, a statistically significant survival benefit was observed in the combination treatment group, and half of the combination group mice survived for the duration of the experiment, whereas there were no surviving mice in any of the other three treatment groups (Figure 6B).

**Figure 7:**
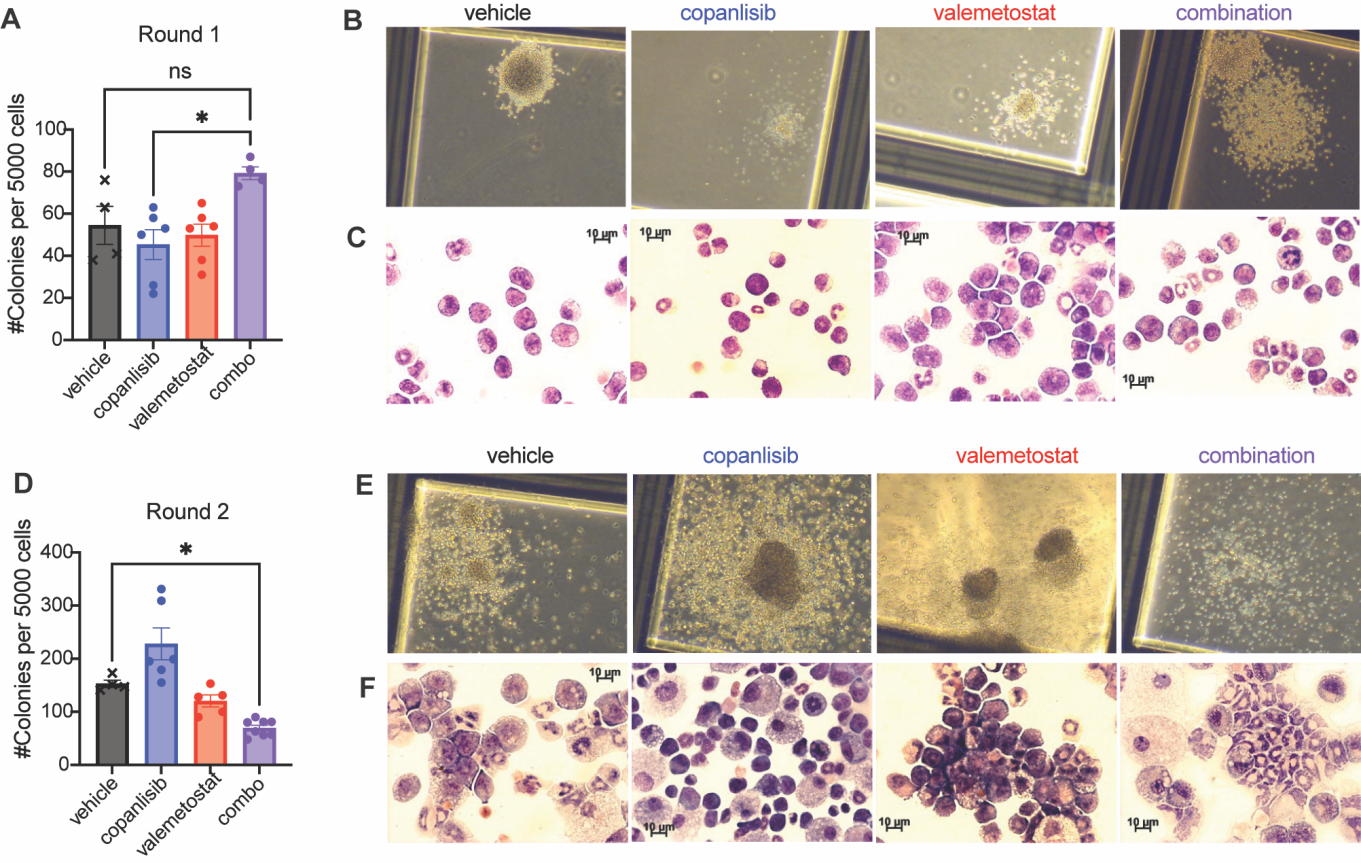
PI3K Inhibition Cooperates with EZH1/2 Dual Inhibition to Target LSCs in NPM1c-NRAS AML **(A)** Results from Round 1 of re-plating assay using splenocytes from moribund mice.**(B-C)** Representative images of colonies formed **(B)** and cytospins made from respective colonies **(C)** from Round 1 of re-plating. **(D)** Results from Round 2 of re-plating assay using splenocytes from moribund mice.**(E-F)** Representative images of colonies formed **(E)** and cytospins made from respective colonies **(F)** from Round 2 of re-plating. Each value is presented at mean +/− standard error of the mean (SEM). One-way ANOVA test with Tukey’s multiple comparisons was used in D and F. *P ≤ 0.05 ns = not significant.

To examine LSC function after drug treatment, we performed serial re-plating assays with splenocytes from drug-treated leukemic mice. We initially observed an overall increase in the number of colonies from combination treated mice (Figure 7A). However, colony and cellular morphology indicated signs of myeloid differentiation, which was less apparent in either single agent group (Figure 7, B and C). Upon re-plating of these colonies, we found that cells from the combination-treated mice had decreased serial replating capacity, with a significant decrease in the number of colonies at Round 2 (Figure 7D), and morphological differences were only visible in the combination group after serial replating (Figure 7, E and F). Together, these data indicate a decrease in the LSC population upon treatment with the combination of copanlisib and valemetostat.

**Figure 8:**
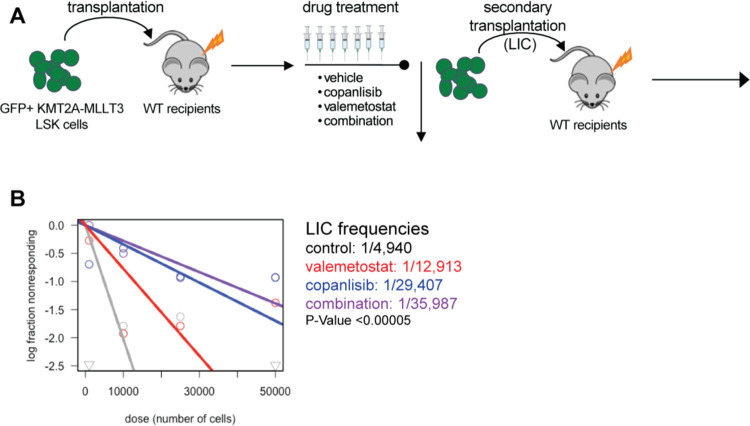
PI3K Inhibition Cooperates with EZH1/2 Dual Inhibition to Target LSCs in vivo **(A)** Experimental schematic for bone marrow transplantation assay of KMT2A-MLLT3 AML cells with in vivo drug treatment with 6mg/kg copanlisib administered i.p. MWF +/− 100mg/kg valemetostat administered oral gavage Daily M-F, followed by secondary transplantation LIC ELDA assay. **(B)** ELDA analysis based on survival data from secondary transplantation with limiting number of GFP+ leukemic cells.

Next, we sought to determine the leukemia initiating cell frequency differences upon treatment with our combination regimen. To test this, we injected sorted LSK cells transduced with the KMT2A-MLLT3-GFP retrovirus into lethally irradiated WT recipients. Once engraftment was confirmed, we randomized the mice by %GFP in the peripheral blood and then treated them for two weeks with 6mg/kg copanlisib intraperitoneally, 100mg/kg valemetostat by oral gavage, or the combination. After two weeks of treatment, we euthanized the animals and performed disease burden analysis (Figure 8A, Supplementary Figure 11). First, to confirm the on target effects of our drug treatments, we performed Western analysis on spleens from the primary transplant recipients. As expected, we observed a decrease in pS6, a downstream effector of the PI3K/AKT/MTOR pathway, in mice treated with copanlisib or with the copanlisib and valemetostat combination (Supplementary Figure 11, A and B). Additionally, we observed a decrease in H3K27me3 in our valemetostat-treated and combination-treated mice, indicating that the drugs were dosed appropriately (Supplementary Figure 11, A and C). Given the aggressive nature of this model, we did not see combinatorial effects on disease burden after two weeks of treatment (Supplementary Figure 11, D-F). However, we did note a significant increase in the frequency of the CD115+Gr1− cell population in each treatment group compared to the untreated controls, consistent with monocytic differentiation (Supplementary Figure 11G).

To determine if we are targeting the leukemia initiating cells with this treatment, we performed limiting dilution secondary transplantation with sorted GFP+ leukemic cells from mice in each treatment group, without any additional treatment in the secondary transplant recipients. As expected, the combination group had prolonged survival with transplantation of every cell dose compared to vehicle control, and at the lowest dose none of the combination treated mice developed leukemia during the observation period (Supplementary Figure 12). Importantly, extreme limiting dilution analysis (ELDA) [44] revealed a statistically significant 10-fold reduction in LIC frequency in our combination-treated mice compared to the untreated group (Figure 8B).

To determine the effects of the copanlisib and valemetostat drug combination on healthy hematopoietic cells, we treated whole bone marrow cells from WT mice in colony assays in methylcellulose supplemented with myeloid growth factors. Although we observed a statistically significant reduction in total colony numbers with our combination treatment (Supplementary Figure 13A), these cells were still able to produce every type of myeloid colony (Supplementary Figure 13B). This suggests that there is likely to be a therapeutic window for this drug combination for the hematopoietic system.

### The copanlisib and valemetostat combination reduces colony formation of AML and MDS patient samples

**Figure 9:**
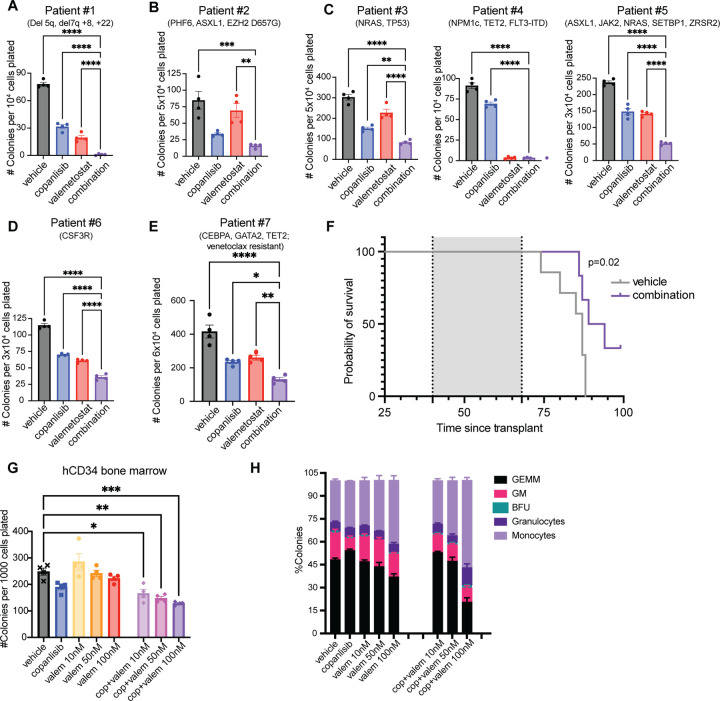
PI3K Inhibition Cooperates with EZH1/2 Dual Inhibition to Impair Colony Formation by AML and MDS Patient Cells **(A)** Colony assays of AML patient sample #1 treated with 100nM copanlisib and 500nM valemetostat **(B)** Colony assay of AML patient sample #2 treated with 100nM copanlisib and 100nM valemetostat. **(C)** Colony assay of AML patient samples #3–5 treated with 100nM copanlisib and 50nM valemetostat. **(D)** Colony assay of AML patient sample #6 treated with 100nM copanlisib and 10nM valemetostat. **(E)** Colony assay of AML patient sample #7 treated with 100nM copanlisib and 50nM valemetostat **(F)** Kaplan-Meier survival curve of PDX using AML patient sample #7 comparing treatment groups. Log-Rank test was used. **(G-H) (G)** Colony counts and **(H)** colony type breakdown on colony assays performed on hCD34+ cells treated with 100nM copanlisib in combination with various doses of valemetostat. Each value is presented as mean +/− standard error of the mean (SEM). One-way ANOVA test with Tukey’s multiple comparisons was used in A-E, and G. ****P ≤ 0.0001 ***P ≤ 0.001 **P ≤ 0.01 *P ≤ 0.05

To determine the clinical relevance of this drug combination in AML, we asked if this treatment regimen can also reduce proliferation of AML and high-risk MDS patient cells. We tested our combination treatment in a variety of primary MDS and AML patient samples in methylcellulose colony assays with myeloid growth factors in the presence of copanlisib, valemetostat, or the combination. Consistent with our cell line data, we observed combinatorial effects of copanlisib and valemetostat on the colony forming capacity of AML patient samples of a variety of molecular and cytogenetic subtypes, compared to either single agent alone (Figure 9A-E; Supplementary Table 1).

Importantly, in several patient samples we observed effects of combination treatment on colony formation with lower doses of valemetostat (10–100nM) than the dose needed to observe a decrease in proliferation in AML cell lines (500nM) (Figure 9, B-D). Furthermore, we observed activity of this drug combination even in difficult to treat AML subtypes, such as AML with complex cytogenetics (Figure 9A), with mutated TP53 (Figure 9C), or with venetoclax resistance (Figure 9E).

Given the promising results in colony assays on patient samples, we tested our combination treatment in a PDX model generated by transplantation of NSG mice with an AML sample from a patient who had relapsed after venetoclax treatment (Patient #7). Upon engraftment, we treated the mice for four weeks and then monitored for survival (Supplementary Figure 13C). Importantly, we observed a significant survival advantage in the PDX mice treated with the copanlisib + valemetostat combination compared to the untreated mice (Figure 9F). Notably, we observed no significant differences in survival in our single treatment groups compared with untreated mice (Supplementary Figure 13D) Furthermore, we found no significant differences in the changes in body weight between treatment groups, indicating minimal toxicity of the combination treatment (Supplementary Figure 13, E and F).

To test the effects of this drug combination on healthy cells and to determine a therapeutic window for this combination treatment, we performed colony assays on healthy donor bone marrow CD34+ cells with copanlisib and valemetostat. We observed a statistically significant reduction in total colony numbers with combination treatment (Figure 9G). However, the degree of inhibition was lower than for most MDS or AML patient samples at the same drug doses, and all myeloid colony subtypes were still present at every dose combination (Figure 9H), suggesting that AML or high-risk MDS cells are more sensitive to this drug combination than healthy human bone marrow CD34+ cells.

## Discussion

Determining more universal therapeutics that can target LSCs and finding ways to leverage the acquired resistance mechanisms to these therapeutics are critical to improve outcomes for AML patients. The PI3K pathway is activated in a variety of cancer types, including AML, and this pathway has been targeted in other forms of cancer with varying degrees of success[25]. Here we report that LSCs in AML have a dependency on PI3Kα. Our results contrast with data recently reported by Gu et al[23], who reported no effects of *Pik3ca* shRNA knockdown in KMT2A-MLLT3 LSCs ex vivo. We believe that our results reflect some differences in experimental approaches between the two studies, since we performed conditional genetic deletion of *Pik3ca* and pharmacologic inhibition of PI3Kα, rather than shRNA knockdown.

While our data in mouse AML models and in patient samples suggest that PI3K inactivation can inhibit AML cell proliferation, our results suggest that single agent PI3K inhibitor activity is likely to be transient, with resistance developing over several days. This could be one of the reasons why prior clinical trials testing single agent pan-PI3K inhibitors in AML had limited efficacy [20, 45]. However, combining multiple classes of inhibitors can allow for improved efficacy compared to therapies targeting a single mechanism[46].

**Figure 10:**
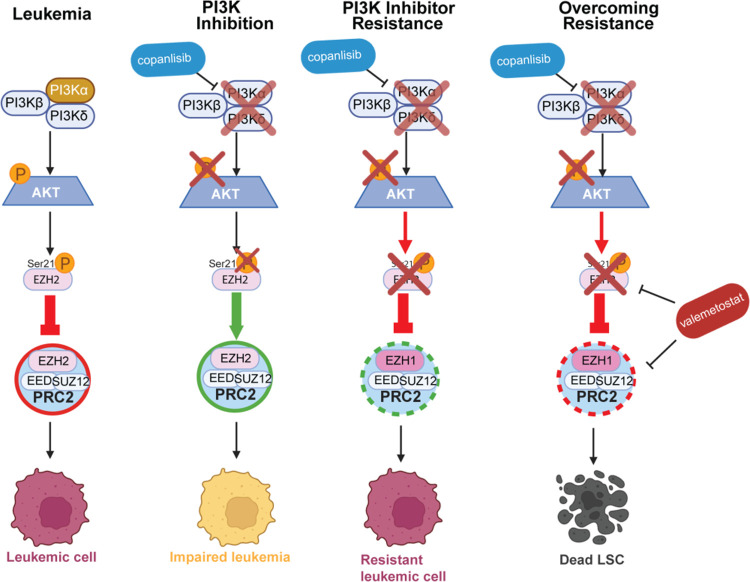
PI3K Inhibition Cooperates with EZH1/2 Dual Inhibition to Overcome Acquired Resistance to PI3K Inhibition Alone Schematic summarizing overall findings. A red circle around PRC2 signifies inhibition of PRC2 function, while green represents de-repression of PRC2 function. Figure created with Biorender.

Here we show that inactivation of PI3Kα is sufficient to inhibit AKT activity in AML cells. We find that this AKT inhibition leads to myeloid differentiation and loss of self-renewal in LSCs through the de-phosphorylation of EZH2 at Ser21 (Figure 10). Consistent with this finding, we observed an increase in H3K27me3 in AML cells treated with the PI3K inhibitor copanlisib. Interestingly, it has been reported that forced overexpression of a myristoylated version of AKT can also result in differentiation of AML cells via FOXO inhibition[47]. This does not necessarily contradict our findings, but rather supports the idea that tight regulation of the PI3K/AKT pathway is critical to maintain normal homeostasis, including in LSCs. Together, these data also suggest that either insufficient or excessive pathway activation can result in impaired LSC maintenance.

We also demonstrate that leukemic cells can develop adaptive resistance to PI3K inhibition via a non-genetic mechanism, whereby EZH2 protein levels are later downregulated to circumvent the effect of PI3K/AKT inactivation on EZH2 phosphorylation (Figure 10). Gollner and colleagues previously reported that proteasomal degradation of EZH2 can be a mechanism of chemotherapy resistance in AML [48]. Our results suggest that loss of EZH2 protein can also give the leukemic cells a survival advantage in the setting of PI3K inactivation. Interestingly, a recent report also identified a similar resistance mechanism with EZH2 downregulation in response to FLT3 inhibitors, but this was limited to FLT3-mutated AML[49].

Our findings add additional context to the contradictory roles of EZH2 in myeloid malignancies. As Basheer and colleagues demonstrated, the role of EZH2 is very context-dependent, with it being either a tumor suppressor or an oncogene depending on the stage of disease and depending on which of its targets are being manipulated by its activity[13]. Somewhat counterintuitively, Fujita et al noted an increase in EZH2 expression in the LSC pool, whereas Basheer et al indicate that EZH2 acts as a tumor suppressor at disease onset. We posit that part of what dictates this context is the phosphorylation status of EZH2 at Ser21. Given these complexities, it follows that the targeting of EZH2 and EZH1/2 in preclinical studies has had mixed results[37, 50]. Notably, Fujita and colleagues demonstrated that the genetic ablation of both EZH1 and EZH2 is required to successfully target leukemic cells and deplete the LSC pool, compared to targeting EZH2 alone[42]. Our results are consistent with their findings.

Several studies have identified non-canonical roles for EZH2 in a variety of cancer types [51–53]. There is evidence that the phosphorylation of EZH2 downstream of PI3K likely has other roles aside from disrupting its histone methyltransferase abilities[52]. This would provide a plausible explanation for why the downregulation of EZH2 protein levels is necessary for the EZH1/2 dual inhibitor to have efficacy in the leukemic setting.

EZH1/2 inhibitors as solo agents have had varying degrees of success in preclinical studies in AML[37, 50]. Our proposed treatment plan introduces a mechanism by which we can increase the efficacy of both PI3K inhibitors and EZH1/2 inhibitors in AML. We also show that we can leverage this resistance mechanism by combining a PI3K inhibitor with an EZH1/2 dual inhibitor, exploiting the new dependency of leukemic cells on EZH1 in the context of PI3K inhibition (Figure 10). This combination is particularly effective at depleting functional LSCs, as we observed in the NPM1c-NRAS and KMT2A-MLLT3 mouse models of AML. Several PI3K inhibitors, including copanlisib (Bayer), were approved by the FDA, though copanlisib was recently removed from the U.S. market due to insufficient efficacy in follicular lymphoma, despite its low toxicity in patients[54]. Although no PI3K inhibitors are currently approved for AML treatment, several PI3K/AKT pathway inhibitors, including the PI3Kα-selective inhibitors alpelisib and inavolisib, are approved for other malignancies and have reasonable toxicity profiles in patients [55, 56]. The EZH1/2 inhibitor valemetostat (Daichi Sankyo) is approved in Japan for the treatment of adult T cell leukemia-lymphoma (ATLL) and was also well-tolerated in patients [54, 57]. While the specific combination of a PI3K inhibitor and valemetostat has not yet been tested in patients, our colony assays on mouse bone marrow cells and healthy donor CD34 cells, and combination drug treatment in multiple mouse AML models suggest that a therapeutic window should be achievable.

In the last decade, there have been advancements in the targeting of epigenetic mechanisms in AML therapies, typically in combination with other therapeutics[3]. By targeting a signaling pathway alongside an epigenetic regulator, we are able to better target a more diverse range of AML subtypes. Our work suggests that a broad range of AML cases may be susceptible to the combination of a PI3K inhibitor and an EZH1/2 inhibitor. However, more studies will be needed to elucidate the specific determinants of response to this drug combination in AML, and to understand which specific molecular or cytogenetic profiles could confer sensitivity to our proposed combination treatment. Importantly, several difficult to treat subtypes of AML, including AML with complex cytogenetics, TP53 mutated AML, and venetoclax-resistant AML cells are still sensitive to the copanlisib and valemetostat combination. In summary, our data demonstrate that combining PI3K inhibitors with EZH1/2 inhibitors may be a promising therapeutic regimen for AML patients, especially for depleting leukemia-initiating cells to reduce the rate of relapse.

## Methods

### Mice

Mice were maintained under pathogen-free conditions in a barrier facility in microisolator cages based on a protocol approved by the Institutional Animal Care and Use Committee at Albert Einstein College of Medicine (AECOM). *Pik3cd* germline KO, *Pik3ca*-lox/lox, and *Pik3cb*-lox/lox were described previously[58]. For *Pik3ca*-lox/lox;*Pik3cb*-lox/lox excision, pIpC (Sigma-Aldrich) was dissolved in Hanks’ balanced salt solution, and 250 μg was injected intraperitoneally three times on nonconsecutive days. Experimental mice evenly included both males and females, and similar findings are reported for both sexes. For transplantation experiments, donor mice were 6–10 weeks old and recipient mice were 6–8 weeks old. Genotypes of each allele (*Pik3ca*, *Pik3cb*, and *Pik3cd*) were determined by PCR using genomic DNA from tails as previously described[58].

### Sex As a Biological Variable

Our study examined both male and female animals and samples from both male and female patients, and similar findings are reported for both sexes.

### Generation of Leukemic Mice

To generate MLL-AF9 leukemic mice, Lineage-Sca1+cKit+ cells were sorted into RPMI media with 10ng/ml IL3, 10ng/ml IL-6, 50ng/ml SCF, 10ng/ml TPO and 20ng/ml Flt3L. Cells were transduced with viral sup generated from the plasmid MSCV-KMT2A-MLLT3-GFP. Transduced cells were plated in methylcellulose for 7 days. 150,000–200,000 sorted GFP+ cells and 250,000 CD45.1+ B6.SJL (The Jackson Laboratory; Strain No. 002014) helper bone marrow cells were transplanted into lethally irradiated (9 Gy) C57Bl/6 (Taconic) recipient mice. To generate the NPM1c/NRAS leukemia model, 250,000–300,000 Npm1^LSL-CA/+;^Nras^LSL-G12D^ frozen bone marrow cells (provided as a gift from Dr. Linde Miles) and 700,000 CD45.1 + B6.SJL helper bone marrow cells were transplanted into lethally-irradiated C57/Bl6 recipient mice. Patient-derived xenotransplantation was performed by transplanting 1,200,000 cells from frozen bone marrow from Patient sample #7 (Supplementary Table 1) into sub-lethally irradiated (2 Gy) NSG mice. Donor cells were injected into the tail-vein and recipient mice were given 100 mg/mL Baytril-100 (Bayer) in drinking water for 4 weeks after transplantation. Mice were euthanized upon signs of development of AML.

### Bone Marrow Aspiration

For femoral bone marrow aspiration mice were anesthetized. The animal’s leg was first disinfected with 70% ethanol, a fine needle was then inserted into the femoral bone marrow cavity through the distal condyles. A small volume (up to 10 uL) of bone marrow was aspirated as this has been shown not to compromise the animal’s functionality of the leg or their overall health. Sides (left/right) were alternately used for sequential aspirates. The animals received 5mg/kg Banamine preemptive per subcutaneous injection as an analgesic.

### Drug Treatment of Mice

The mice were treated 6mg/kg copanlisib three times per week on alternating days intraperitoneally, and with 100mg/kg valemetostat five days a week by oral gavage, or with a combination of both drugs. The control mice were treated with solvents of respective drugs: copanlisib vehicle: PEG400/acidified water, pH 3.5–4 intraperitoneally (3 times per week) or valemetostat vehicle: 0.5% MC Solution by oral gavage 5 times per week.

### Cell culture

NOMO1, MOLM13, MOLM14, and HEL cells were cultured in RPMI with 10% fetal bovine serum (FBS) and 1% Penicillin-Streptomycin (P/S). The venetoclax resistant cell line MOLM13-VR was generated as previously described [59]. OCI-AML3 cells were cultured in α-MEM with 20% FBS and 1%P/S. All cells were kept in a 5% CO_2_ 37°C incubator. Cell lines were authenticated with STR profiling with ATCC and DSMZ databases by the Albert Einstein genomics core facility and verified to be free of mycoplasma on a monthly basis.

### Plasmids

The MSCV-KMT2A-MLLT3-GFP and MSCV-KMT2A-MLLT3-NEO plasmids were a gift from Scott Armstrong’s lab. pcDNA3-3myc-6His-EZH2 21A and pcDNA3-3myc-6His-EZH2 21D were a gift from Mien-Chie Hung (Addgene plasmids # 42663 and # 42664). The cDNA from each EZH2 mutant plasmid was subcloned into pMSCV-IRES-mCherry FP, which was a gift from Dario Vignali (Addgene plasmid # 52114). MSCV-EZH2-PGK-Puro-IRES-GFP was a gift from Christopher Vakoc (Addgene plasmid # 75125). The Cre-ER puro plasmid was a gift from the Gilliland lab.

### Colony Forming Assays

Cells were plated in 35x10mm dishes of Mouse Methylcellulose Complete Media (R&D HSC007). For ex vivo excision of PI3K alleles in MSCV-Cre-ER-PURO transduced colonies, a 4-Hydroxytamoxifen (4-OHT) stock was made in ethanol, and then dissolved in HSC007 methylcellulose at a final concentration of 20nM. Dishes were incubated at 37C for seven days. Colonies were manually counted under an inverted microscope (Olympus CKX41). Cells were then washed off the plate with PBS. A portion of the cells were prepared for microscopy using Cytofuge2 Cytocentrifuge (StatSpin) cytospin and stained with Wright-Giemsa stain (Fisher Scientific 22-122911). Higher resolution images were acquired using a Zeiss Axiovert 200M Microscope with a digital camera. Image acquisition was performed using AxioVision software.

### Human Patient Sample Assays

Healthy human CD34+ BM cells were purchased from Stem Cell Technologies and stored at −150C. Experiments with patient samples were approved under the Albert Einstein College of Medicine Institutional Review Board IRB protocol #2015-4600. De-identified patient samples were obtained with written informed consent through the Albert Einstein College of Medicine biobank IRB protocol #2005-536, in accordance with the Declaration of Helsinki. All patient and BM donor characteristics are listed in Table S1. The samples were defrosted in RPMI 1640, washed, resuspended in RPMI 1640/FBS (fetal bovine serum) media (20% FBS, 1% penicillin-streptomycin) and cultured for 1 hour at 37C. Cells were passed through a 30-μm cell strainer to remove dead cell clumps, washed with RPMI 1640, and resuspended in IMDM +2%FBS media. Cells were plated in quadruplicate in MethoCult H4435 Enriched (StemCell Technologies) at single cell density at indicated concentrations ranging from 1−5x10^4^ cells/plate. Colonies were counted manually after 10–12 days in culture.

### Microarray Analysis and GSEA

The GFP+ GMPs were sorted from the bone marrow of transplant recipient mice following the gating strategy in Fig S3D using the Aria II instrument (BD Biosciences). Cells were sorted into 50μl RNA extraction buffer (ARCTURUS PicoPure RNA Isolation Kit (Life Technologies, Invitrogen) and stored at −80°C. RNA was isolated using the (ARCTURUS PicoPure RNA Isolation Kit (Life Technologies, Invitrogen) according to the manufacturer’s instructions. RNA quantification was performed using the RNA Quantification Kit for SYBR Green I and ROX^™^ Passive Reference Dye (Thermo Fisher, catalog no. 902905). Total RNA was amplified and hybridized to the Gene Chip ^®^ Mouse Transcriptome Pico Assay 1.0 (Affymetrix part number: 902663) using the GeneChip^™^ Hybridization, Wash, and Stain Kit (catalog no. 900720). Raw data was analyzed for quality control using Expression Console software (Affymetrix). After quality control, microarray data was analyzed by Gene Set Enrichment Analysis (GSEA) using MSigDB software (http://software.broadinstitute.org/gsea/index.jsp), Transcriptome Analysis Software (Affymetrix), and Gene Ontology Enrichment analysis (Gene Ontology Consortium; geneontology.org). The microarray data is available at the GEO Expression Omnibus under accession number GSE261355.

### Cell Proliferation Assays

Cells were plated in four 96-well plates (1 x 10^5^ cells/well) and treated with drugs in triplicate. Drugs and media were replenished every 3 days. At 0 hours, 24 hours, 48 hours, 72 hours, 6 days, 9 days, and 12 days the amount of ATP present was measured using CellTiter-Glo Luminescent Cell Viability Assay (Promega, Catalog no. G7570). Cell Titer Glo reagent was added 1:1 before allowing the plate to gently rock 20 minutes at room temperature. Luminescence measured by a PerkinElmer Victor X5 Multilabel Plate Reader.

### Flow Cytometry

For flow cytometry analysis of stem and progenitor cell populations, bone marrow cells were stained with a lineage cocktail of species-appropriate biotin-labeled lineage antibodies for 30 min at 4C, followed by species appropriate fluorochrome-conjugated surface antibodies for 20 min at 4C (Table S2-4). For non-stem and progenitor flow analysis, cells were stained with species-appropriate fluorochrome-conjugated surface antibodies for 20 min at 4C (Table S2-4). For intracellular flow analysis, cells were stained with all surface antibodies as described above, and then fixed with cytofix/cytoperm solution (BD biosciences BDB554714) according to the manufacturers protocol. Cells were then stained with either fluorochrome-conjugated intracellular antibodies or unconjugated primary antibodies (Table S3) for 30 min at room temperature, followed by species appropriate fluorochrome-conjugated secondary antibody for 30 min at room temperature. Flow sorting was performed on BD FACSAria III. Flow cytometry analysis was performed on the BD FACS LSRII or Cytek Aurora. Cell cycle analysis was performed with Ki-67 and Hoechst staining. Apoptosis analysis was performed using an APC Annexin V with 7-AAD kit (BD Biosciences), following the manufacturers protocol. Analysis of all flow cytometry data was performed using FlowJo software (version 10).

### Time Course Drug Treatment

MOLM14 or NOMO1 cells were treated with 1μM alpelisib (BYL719; S2814 Selleckchem), 1 μM buparlisib (BKM120; S2247 Selleckchem), 100nM or 500nM Copanlisib (BAY 90-6946; S2802 Selleckchem) or DMSO control and harvested at 3 hour, 24 hour, and 48 hour time points. Cell pellets were stored at −80°C.

### Western Blotting

Protein was extracted from frozen cells with Pierce IP Lysis Buffer and Halt Protease/Phosphatase Inhibitor Cocktail (Thermo Fisher), 30uL/10^6^ cells, after PBS wash. Sonication was used to extract nuclear proteins. Lysate Protein concentration was determined using a Bradford assay with Bio-Rad Protein Assay Dye Reagent. Proteins were separated with SDS-PAGE on a NuPAGE Bis-Tris 10% polyacrylamide gel (Thermo Fisher) or a Novex Tris-Glycine 10–20% polyacrylamide gel and transferred to a 0.2um nitrocellulose membrane. The membranes were blocked in Tris-Buffered Saline 0.1% Tween 20 (TBST) with 5% bovine serum albumin (BSA) and washed with TBST. The membranes were incubated overnight at 4°C with primary antibodies as listed in Table S5. Internal loading controls were incubated 1 hour at room temperature. After washing, the membrane was blotted with fluorescent secondary antibodies for 1 hour. Bands were visualized using the Odyssey Fc imaging system (LI-COR) and protein band quantification was measured using Image Studio (version 5.2.5). Please refer to Table S5 for all Western antibody information. Some membranes were stripped with ReBlot Plus Strong antibody stripping solution (Millipore #2504) and then re-blocked and re-probed.

### RT-PCR

RNA was isolated from frozen cell pellets using the Qiagen RNeasy and Qiashredder kits per manufacturer’s instructions. cDNA was made from isolated RNA using RNA to cDNA EcoDry Premix (Random Hexamers) (Takara #639546) as per manufacturer’s instructions. PCR was performed using SYBR-Green reagents and acquired on the Viia7 Real-Time PCR system. For primer sequences see Table S6.

### Histone extraction and digestion

Histone proteins were extracted from the pellet as described by [60] to ensure good-quality identification and quantification of single histone marks. Briefly, histones were acid-extracted with chilled 0.2 M sulfuric acid (5:1, sulfuric acid : pellet) and incubated with constant rotation for 4 h at 4°C, followed by precipitation with 33% trichloroacetic acid (TCA) overnight at 4°C. Then, the supernatant was removed and the tubes were rinsed with ice-cold acetone containing 0.1% HCl, centrifuged and rinsed again using 100% ice-cold acetone. After the final centrifugation, the supernatant was discarded and the pellet was dried using a vacuum centrifuge. The pellet was dissolved in 50 mM ammonium bicarbonate, pH 8.0, and histones were subjected to derivatization using 5 μL of propionic anhydride and 14 μL of ammonium hydroxide (all Sigma Aldrich) to balance the pH at 8.0. The mixture was incubated for 15 min and the procedure was repeated. Histones were then digested with 1 μg of sequencing grade trypsin (Promega) diluted in 50mM ammonium bicarbonate (1:20, enzyme:sample) overnight at room temperature. Derivatization reaction was repeated to derivatize peptide N-termini. The samples were dried in a vacuum centrifuge.

### Sample desalting

Prior to mass spectrometry analysis, samples were desalted using a 96-well plate filter (Orochem) packed with 1 mg of Oasis HLB C-18 resin (Waters). Briefly, the samples were resuspended in 100 μl of 0.1% TFA and loaded onto the HLB resin, which was previously equilibrated using 100 μl of the same buffer. After washing with 100 μl of 0.1% TFA, the samples were eluted with a buffer containing 70 μl of 60% acetonitrile and 0.1% TFA and then dried in a vacuum centrifuge.

### LC-MS/MS Acquisition and Analysis

Samples were resuspended in 10 μl of 0.1% TFA and loaded onto a Dionex RSLC Ultimate 300 (Thermo Scientific), coupled online with an Orbitrap Fusion Lumos (Thermo Scientific). Chromatographic separation was performed with a two-column system, consisting of a C-18 trap cartridge (300 μm ID, 5 mm length) and a picofrit analytical column (75 μm ID, 25 cm length) packed in-house with reversed-phase Repro-Sil Pur C18-AQ 3 μm resin. Peptides were separated using a 30 min gradient from 1–30% buffer B (buffer A: 0.1% formic acid, buffer B: 80% acetonitrile + 0.1% formic acid) at a flow rate of 300 nl/min. The mass spectrometer was set to acquire spectra in a data-independent acquisition (DIA) mode. Briefly, the full MS scan was set to 300–1100 m/z in the orbitrap with a resolution of 120,000 (at 200 m/z) and an AGC target of 5x10e5. MS/MS was performed in the orbitrap with sequential isolation windows of 50 m/z with an AGC target of 2x10e5 and an HCD collision energy of 30.

Histone peptides raw files were imported into EpiProfile 2.0 software [61]. From the extracted ion chromatogram, the area under the curve was obtained and used to estimate the abundance of each peptide. In order to achieve the relative abundance of post-translational modifications (PTMs), the sum of all different modified forms of a histone peptide was considered as 100% and the area of the particular peptide was divided by the total area for that histone peptide in all of its modified forms. The relative ratio of two isobaric forms was estimated by averaging the ratio for each fragment ion with different mass between the two species. The resulting peptide lists generated by EpiProfile were exported to Microsoft Excel and further processed for a detailed analysis.

Proteomics data submitted to ProteomeXchange via the PRIDE database, accession #: PXD050834.

### Valemetostat Synthesis


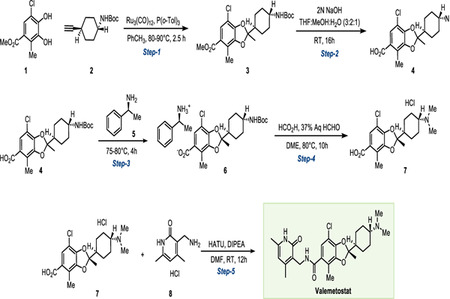


The synthetic procedure was adapted from patent WO/2022/009911.

#### Methyl2-((1*r*,4*r*)-4-((*tert*-butoxycarbonyl)amino)cyclohexyl)-7-chloro-2,4-dimethylbenzo[*d*][1,3]dioxole-5-carboxylate (3).

In a pear-shaped flask equipped with a condenser and a PTFE-coated stirring bar, a mixture of methyl 5-chloro-3,4-dihydroxy-2-methylbenzoate **1** (8 g, 1.0 equiv., 0.04 mol, Accela, cat. # SY291378), tri-*o*-tolylphosphine (3 g, 0.3 equiv., 0.33 mol, 1PlusChem, cat. # 1P003689) and triruthenium dodecacarbonyl (2 g, 0.1 equiv., 4 mmol 1PlusChem, cat. #1P0039U2) was purged with argon/vacuum cycles (4 cycles), then toluene (84 mL) was added. The system was purged with four additional argon/vacuum cycles, and the reaction was then stirred at 120° C for 30 minutes. A mixture of *tert*-butyl (1*r*,4*r*)-4-ethynylcyclohexyl) carbamate **2** (9 g, 1.2 equiv., 0.04 mol, 1PlusChem, cat. #1P00IIXA) in toluene (70 mL) was added to the dark mixture, and the resulting orange solution was stirred for another 2 hours at 120° C. Upon reaction completion (confirmed by TLC), the solvent was evaporated under rotary evaporation, and the crude residue was purified by flash column chromatography (Teledyne CombiFlash Nextgen 300+, SiliaSep PREMIUM cartridge, 10 to 15 % EtOAc in hexane) to afford compound **3** as a pale green semi-solid (13.5 g, 68%). ^**1**^**H NMR** (300 MHz, CDCl_3_) δ 7.53 (s, 1H), 4.37 (s, 1H), 3.84 (s, 3H), 3.39 (s, 1H), 2.38 (s, 3H), 2.07 (dd, *J* = 9.3, 5.6 Hz, 2H), 1.99 – 1.92 (m, 2H), 1.90 – 1.76 (m, 1H), 1.62 (s, 3H), 1.43 (s, 9H), 1.38 – 1.28 (m, 2H), 1.18 – 1.02 (m, 2H).

#### 2-((1*r*,4*r*)-4-((*tert*-Butoxycarbonyl)amino)cyclohexyl)-7-chloro-2,4-dimethylbenzo[*d*][1,3]dioxole-5-carboxylic acid (4).

In a round-bottom flask equipped with a PTFE-coated stirring bar, compound **3** (13 g,1.0 equiv., 30 mmol) was dissolved in THF (106 mL) and MeOH (56 mL), then 2M aqueous NaOH (35 mL) was added. The reaction was stirred at room temperature for 16 hours. Upon completion (confirmed by TLC), the organic solvent was removed by rotary evaporation, and the crude was treated with 2N HCl to adjust pH to 2–3. Water (200 mL) was added to the reaction mixture, causing precipitation of copious solid, which was collected by filtration over a Buckner funnel, washed with 250 mL of hexane), and dried overnight under vacuum compound **4** as an off-white solid (11 g, 87%). ^**1**^**H NMR** (300 MHz, CDCl_3_) δ 7.59 (s, 1H), 4.42 (s, 1H), 3.39 (s, 1H), 2.38 (s, 3H), 2.07 (d, *J* = 11.9 Hz, 2H), 2.00 – 1.91 (m, 2H), 1.83 (tt, *J* = 12.1, 3.2 Hz, 1H), 1.61 (s, 3H), 1.44 (s, 9H), 1.30 (t, *J* = 12.5 Hz, 2H), 1.10 (q, *J* = 12.3 Hz, 2H). **MS**: *m/z* calculated for: C_21_H_28_ClNO_6_ [M+H-Boc]^+^: 325.79 found: 325.79,

#### (*S*)-1-Phenylethan-1-aminium(*R*)-2-((1*r*,4*R*)-4-((*tert*-butoxycarbonyl)amino)cyclohexyl)-7-chloro-2,4-dimethylbenzo[*d*][1,3]dioxole-5-carboxylate (6).

##### Step 1.

In a round-bottom flask equipped with a PTFE-coated stirring bar, **4** (10.5 g, 1 equiv., 24.7 mmol) in 105 mL of 1,2-dimethoxyethane (DME) was stirred at 75–80 °C. (*S*)-1-Phenylethan-1-amine **5** (3.6 mL, 1.13 equiv., 27.9 mmol, Sigma-Aldrich, cat. # 8070470010) was added in one portion, then the reaction mixture was stirred at 80 °C for 4 hours. A mixture of DME (47 mL) and H_2_O (17 mL) was heated to 60 °C was added to the reaction mixture, and the reaction was allowed to cool to room temperature, causing precipitation of a solid. The precipitate was filtered out and washed with DME (200 mL), then dried under vacuum to obtain partially enriched compound **6** as an off-white solid (3.5 g).

##### Step 2.

In a round-bottom flask equipped with a PTFE-coated stirring bar, **6** (3.5 g) was added to a mixture of DME (47 mL) and water (16 mL). To the solution, 1.12 mL of 5M HCl was added at room temperature. After 10 minutes, the reaction mixture was heated to 75 °C, and a solution of (*S*)-1-phenylethan-1-amine (0.72 mL, Sigma-Aldrich, cat. # 8070470010) in DME (5.5 mL) was added dropwise over 10 minutes. The reaction was stirred at 80 °C for 2 hours, then cooled 0 °C, causing the precipitation of a solid. The solid was collected by filtration and washed with DME (100 mL) to afford enriched compound **6** as an off-white solid (2.6 g, 25% over 2 steps). ^**1**^**H NMR** (600 MHz, CD_3_OD) δ 7.47 – 7.38 (m, 5H), 7.09 (s, 1H), 4.41 (q, *J* = 6.9 Hz, 1H), 3.29 – 3.24 (m, 1H), 2.28 (s, 3H), 1.96 (tq, *J* = 9.0, 3.3 Hz, 4H), 1.83 (tt, *J* = 12.0, 3.0 Hz, 1H), 1.61 (d, *J* = 6.8 Hz, 3H), 1.59 (s, 3H), 1.43 (s, 9H), 1.35 – 1.27 (m, 2H), 1.23 – 1.15 (m, 2H). The ^1^H NMR spectrum closely matches the literature report (WO/2022/009911)

#### (*R*)-7-Chloro-2-((1*r*,4*R*)-4-(dimethylamino)cyclohexyl)-2,4-dimethylbenzo[*d*][1,3]dioxole-5-carboxylic acid hydrochloride (7).

In a round-bottom flask equipped with a PTFE-coated stirring bar, to a stirred solution of **6** (2.53 g, 1.0 equiv., 5.90 mmol) in DME (2.53 mL) was added formic acid (5.04 mL, 22.5 equiv., 134.00 mmol, Fluka, cat. #60–006-16) and 37% aqueous formaldehyde (3.75 mL, 17.1 equiv., 102.00 mmol, Thermo-Fisher, cat. #119690010) under a nitrogen atmosphere. The reaction mixture was stirred at 75–80 °C for 10 hours, then upon completion (confirmed by LC-MS) the solvent was removed under rotary evaporation to afford the crude product, which was purified by reverse-phase flash column chromatography (Teledyne CombiFlash Nextgen 300+, SiliaSep PREMIUM C18 cartridge - 40 g, 100% MeCN + 0.01% formic acid to 100% H_2_O + 0.01% formic acid), the compound eluted in 20% MeCN in H_2_O. Fractions containing the product were dried by rotary evaporation and then lyophilized to afford the **7** as an off-white solid (1.4 g, 67%) ^**1**^**H NMR** (600 MHz, CD_3_OD) δ 7.37 (s, 1H), 3.20 (tt, *J* = 12.2, 2.6 Hz, 1H), 2.83 (s, 6H), 2.35 (s, 3H), 2.19 – 2.10 (m, 4H), 2.04 – 1.97 (m, 1H), 1.66 (s, 3H), 1.56 (tt, *J* = 12.9, 6.6 Hz, 2H), 1.42 (tdd, *J* = 16.0, 7.3, 4.0 Hz, 2H). **MS**: *m/z* calculated for: C_18_H_25_ClNO_4_ [M+H]^+^: 354.15 found: 354.10.

#### Valemetostat.

In a round-bottom flask equipped with a PTFE-coated stirring bar, **7** (1.25 g, 1.0 equiv., 3.53 mmol), **8** (1.33 g, 2.0 equiv., 7.10 mmol, 1pluschem, cat #: 1P000EJW) and HATU (2.68 g, 2.0 equiv., 7.01 mmol, 1PlusChem, cat. # 1P001LPW) were dissolved in DMF (12 mL), then DIPEA (6.31 mL, 10.0 equiv., 35.00 mmol, Sigma-Aldrich, cat. # D125806) was added under argon atmosphere, and the reaction mixture was stirred at room temperature for 10 hours. Upon completion of the reaction (confirmed by LC-MS), the reaction mixture was basified with 0.1 N aqueous NaOH to pH 10–11, then the reaction mixture was extracted three times with EtOAc (200 mL each time), the combined organic layers were dried over Na2SO4 and concentrated under rotary evaporation. The obtained crude was dissolved in 150 mL of water and stirred overnight, causing the precipitation of a solid which was collected by filtration and dried under vacuum.The obtained solid was washed once with 1M aqueous NaHCO_3_ (200 mL) and solid-extracted three times with DCM: MeOH (9:1, 200 mL each time). The organic layer was dried over Na_2_SO_4_ and concentrated under rotary evaporation, then the obtained compound was dissolved in a minimal amount of MeCN:H_2_O (3:7) and lyophilized to afford the valemetostat as a white solid (1.28 g, 74.2%) ^**1**^**H NMR** (600 MHz, DMSO-*d*_*6*_) δ 11.47 (s, 1H), 8.12 (t, *J* = 5.0 Hz, 1H), 6.85 (s, 1H), 5.86 (s, 1H), 4.22 (d, *J* = 5.0 Hz, 2H), 3.32 (s, 2H), 2.24 – 2.01 (m, 16H), 1.92 – 1.78 (m, 5H), 1.60 (s, 3H), 1.22 – 1.10 (m, 4H). ^**13**^**C NMR** (151 MHz, DMSO*-d*_*6*_) δ 167.03, 163.43, 149.93, 147.35, 144.01, 143.23, 132.03, 123.23, 121.98, 121.35, 116.25, 109.02, 107.81, 63.04, 45.87, 41.71, 35.51, 27.52, 25.59, 25.57, 22.40, 19.36, 18.65, 12.45. **MS**: *m/z* calculated for: C_26_H_34_ClN_3_O_4_ [M+H]+:488.03. found: 488.30, Purity (HPLC-UV 254 nm): >95%. (*t*_R_= 3.91 min).

The following method was used to check the enantiomer purity:

Column: Daicel, CHIRALCEL OZ-H, 4.6 mm ID X 250 mm L

Elution solvent: n-hexane: ethanol: diethylamine = 60:40:0.04 (v/v)

Flow rate = 1 mL/min, HPLC, detection at 254 nm

Temperature: 25°C, the major isomer eluted at t_R_ = 5.97 min and its retention time closely matches the literature report (US20170073335A1).

#### Reagents

Please refer to Tables S2-5 for the list of Western blot antibodies and flow cytometry antibodies used. All other reagents are listed in their respective methods section.

#### Study approval

All mouse breeding and animal experiments were approved by the Institutional Animal Care and Use Committee under protocol nos. 20170205, 20170206, 00001165, and 00001181. Experiments with patient samples were approved under IRB#2015–4600 and #2005–536.

#### Statistics

GraphPad Prism 10 was used for all statistical analysis. For the comparison of two experimental groups, an unpaired t-test was used. For the comparison of more than two groups, ANOVA test was used with Tukey’s multiple comparisons test. In all graphs, error bars indicate mean ± SEM. A *P* value less than 0.05 was considered significant (*), with 0.01 (**), 0.001 (***), and 0.0001 (****) representing higher levels of significance.

## Supplementary Material

Supplement 1

Supplement 2

Supplement 3

Supplement 4

Supplement 5

Supplement 6

Supplement 7

## Data Availability

The microarray data is available at the GEO Expression Omnibus under accession number GSE261355. Proteomics data submitted to ProteomeXchange via the PRIDE database, accession #: PXD050834.
