# Supplementary material for: Exploiting an Epigenetic Resistance Mechanism to PI3 Kinase Inhibition in Leukemic Stem Cells
Source: bioRxiv. 2025 Jul 15:2025.07.11.663968. Preprint. [Version 1] doi: 10.1101/2025.07.11.663968 (PMC12338508; doi:10.1101/2025.07.11.663968)
Supplement: Supplement 1 [file media-1.pdf]

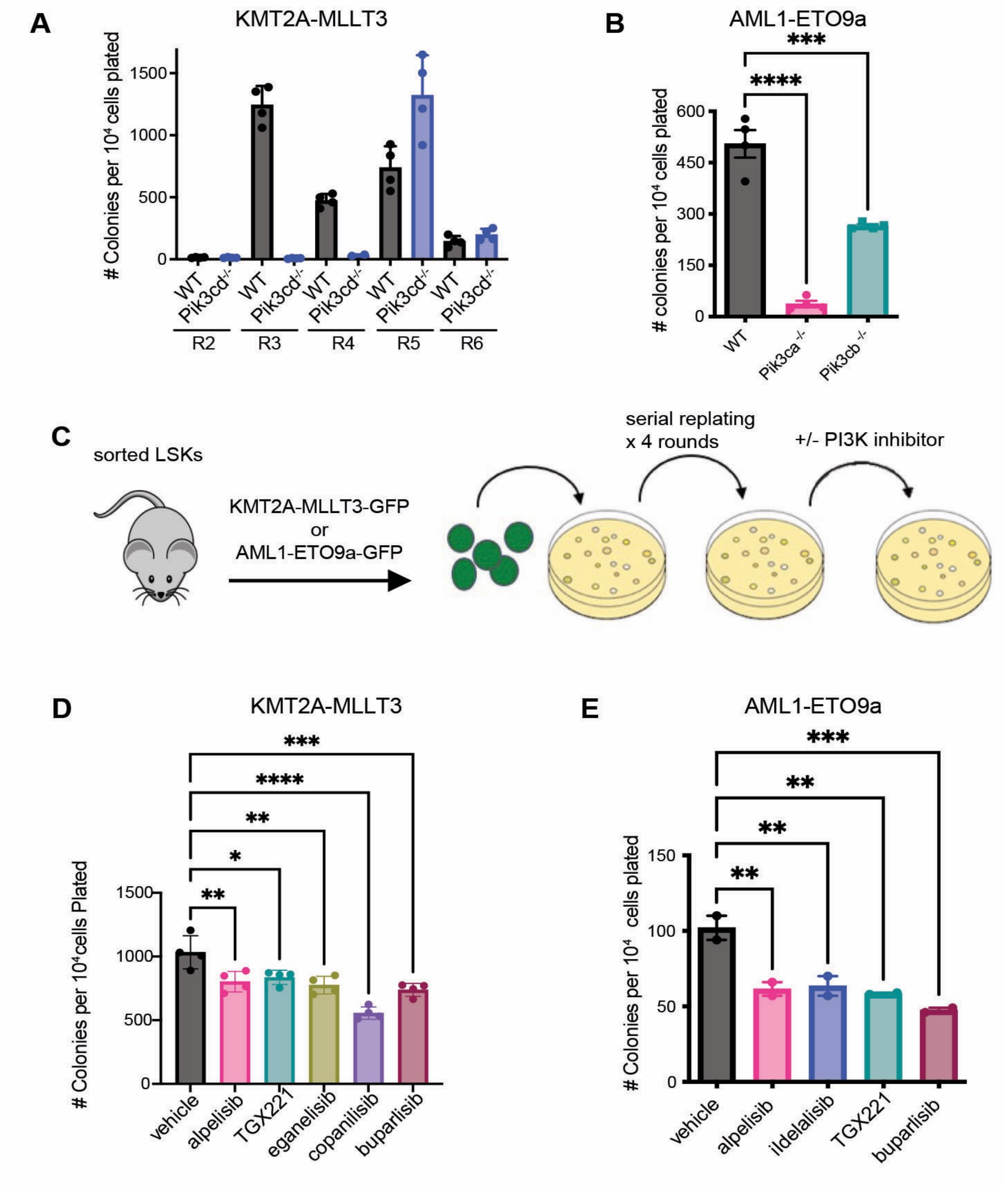

Supplementary Figure 1: PI3K Disruption Promotes Myeloid Differentiation in AML murine models (A) Serial replating results of KMT2A-MLLT3 cells with PI3K δ KO and WT. **(B)** AML1-ETO9a leukemic stem cell colonies after 4 weeks of serial re-plating at one week after acute excision of PI3K isoforms (n=4). **(C)** Experimental schematic for generation of AML1-ETO9a or KMT2A-MLLT3 LSCs treated with PI3K isoform inhibitors. **(D-E)** Leukemic stem cell colony counts in the presence of PI3K inhibitors normalized to vehicle control (alpelisib = α selective, idelalisib = δ selective, copanlisib = α/δ selective, TGX221 = β selective, Eganelisib = γ selective, buparlisib = pan-PI3K inhibitor) **(D)** KMT2A-MLLT3 **(E)** AML1-ETO9a. One-way ANOVA test with Tukey's multiple comparisons was used in B, D, and E. ****P $\leq$ 0.0001 ***P $\leq$ 0.001 **P $\leq$ 0.01 *P $\leq$ 0.05 Each value is presented as mean +/- standard error of the mean (SEM).

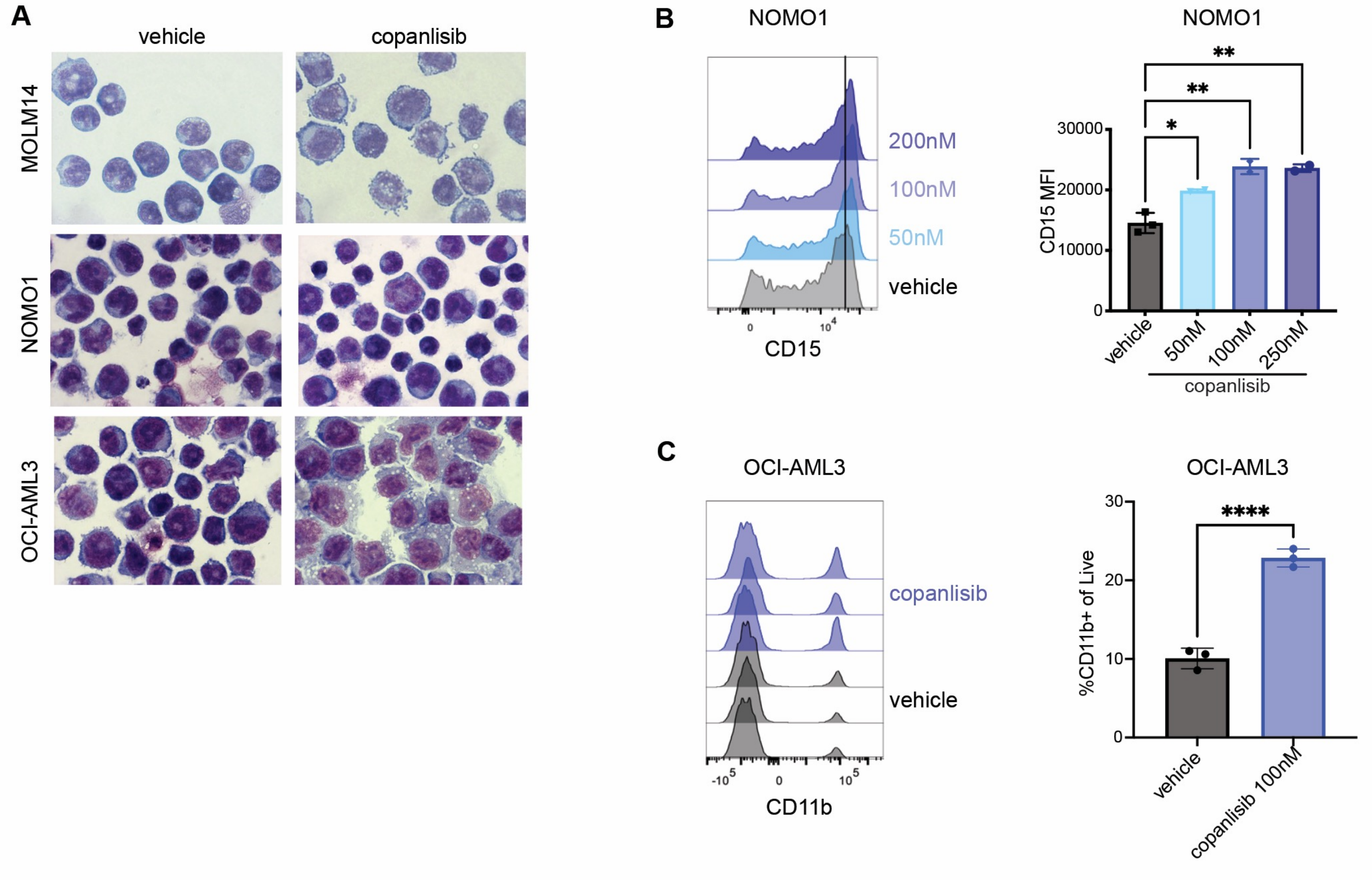

Supplementary Figure 2: PI3K Disruption Promotes Myeloid Differentiation in AML Cell Lines (A) Representative images of cytospins from AML cell lines treated with 100nM copanlisib for 3 days captured with 630x objective **(B)** Histograms and quantification of CD15 expression on NOMO1 cells treated with 100nM copanlisib for 3 days. One-way ANOVA test with Tukey's multiple comparisons was used. $^{**}P \leq 0.01$ $^{*}P \leq 0.05$ **(C)** Representative flow plots and quantification of CD11b expression on OCI-AML3 cells treated with 100nM copanlisib for 3 days. Unpaired t-test was used. $^{****}P \leq 0.0001$ Each value is presented as mean +/- standard error of the mean (SEM).

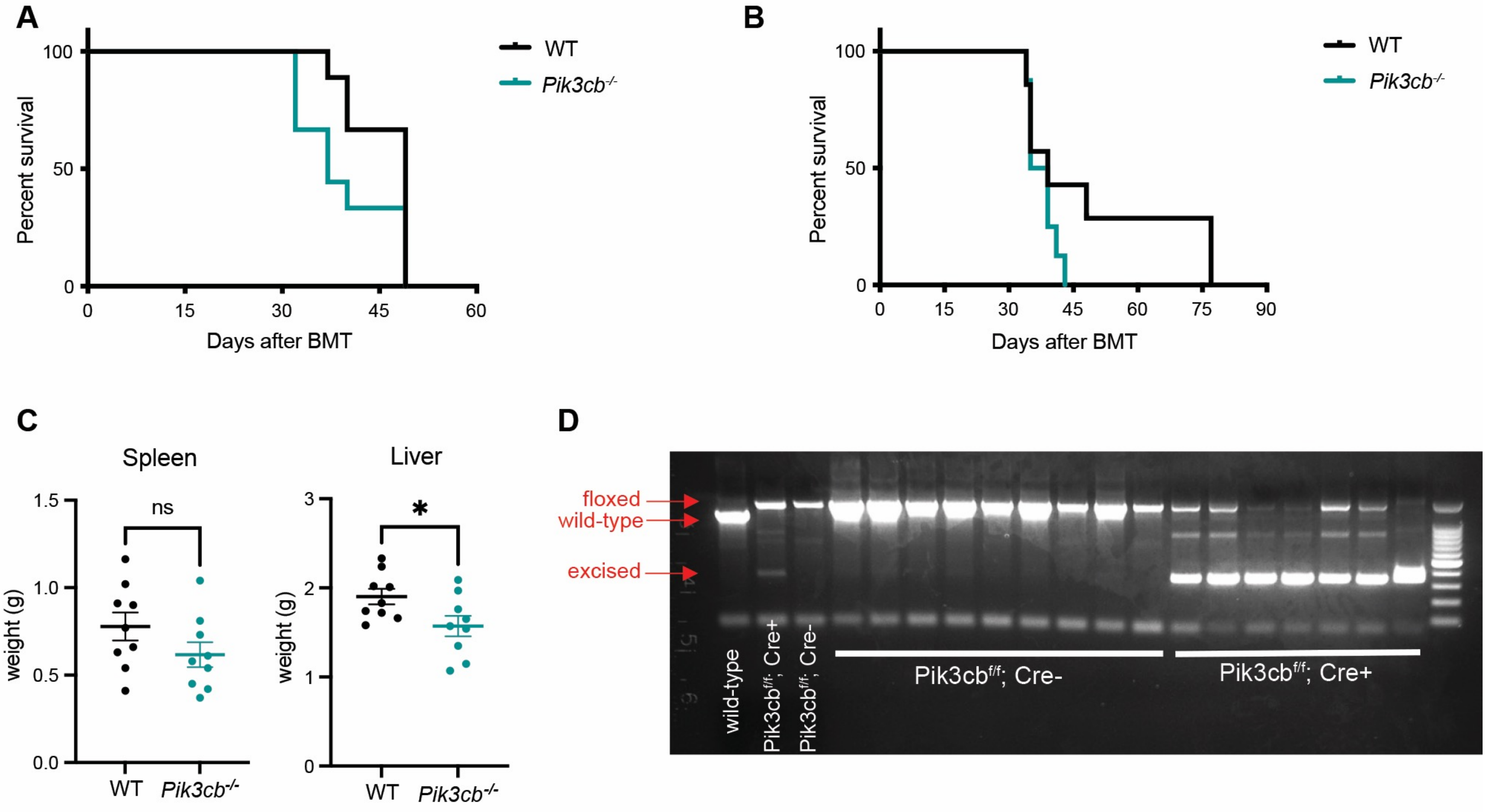

Supplementary Figure 3: p110β is not required for KMT2A-MLLT3 AML progression or LIC activity *in vivo*
(A) Kaplan-Meier survival curve of primary bone marrow transplant (BMT) mice injected with p110β-lox-lox;Mx1-Cre (p110β-/-) or Cre- control (WT) LSK cells transduced with KMT2A-MLLT3 GFP (see Figure 2A). Log-rank analysis was used. **(B)** Kaplan-Meier survival curve of secondary BMT recipients injected with 10,000 KMT2A-MLLT3 GFP+ leukemic cells from p110β-/- or WT primary recipient mice. Log-rank analysis was used. **(C)** Spleen and liver weights from primary BMT mice. Each value is presented as mean +/- standard error of the mean (SEM). Unpaired t-test was used. *P ≤ 0.05
(D) Genotyping results for primary transplant recipients post pIpC injection showing excision of P110β in leukemic cells from Mx1-Cre+ animals

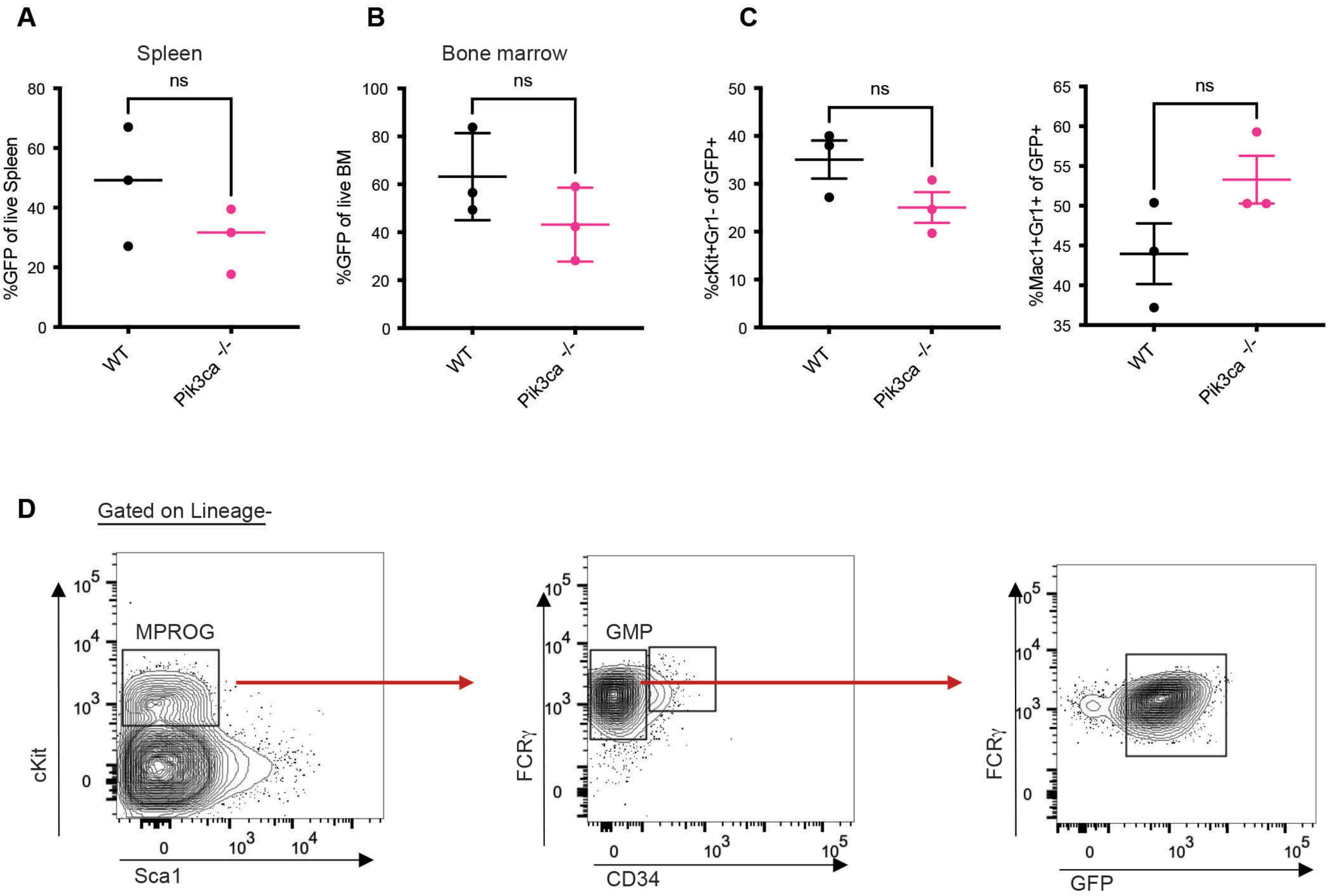

Supplementary Figure 4: There is no significant difference in the disease phenotype at time of death between WT and *Pik3ca*$^{-/-}$ leukemic mice (A) Quantification of %GFP of live spleen cells in leukemic mice **(B)** Quantification of %GFP of live bone marrow cells in leukemic mice. **(C)** Quantification of various populations within the leukemic GFP+ compartment in the bone marrow of leukemic mice. Each value is presented as mean+/- standard error of the mean (SEM). Unpaired t-test $*P \leq 0.05$ **(D)** Gating strategy for LSCs

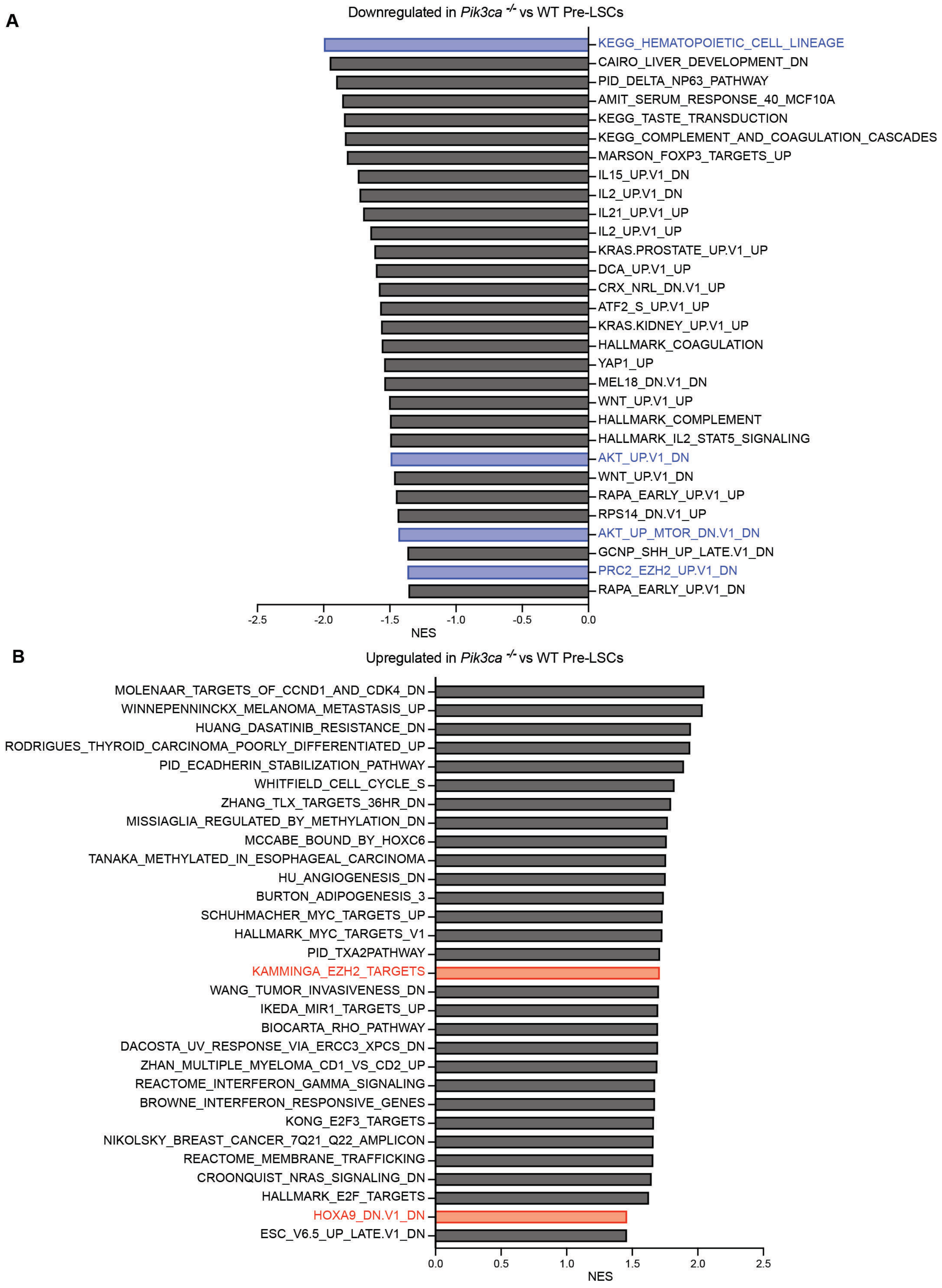

Supplementary Figure 5: *Pik3ca* Deletion Alters pre-LSC Gene Expression Signatures, Including in Hematopoietic Lineage specification and PRC2 regulation

Gene set enrichment analysis (GSEA) was performed on the microarray dataset from preclinical LSCs (pre-LSCs: GFP+ GMPs) sorted from KMT1A-AF9 recipient bone marrow at 45 days post-transplantation. Cutoff of p-value <0.05 and lowest 30 FDR-q values depicted **(A)** Negatively enriched GSEA gene sets **(B)** Positively enriched GSEA gene sets

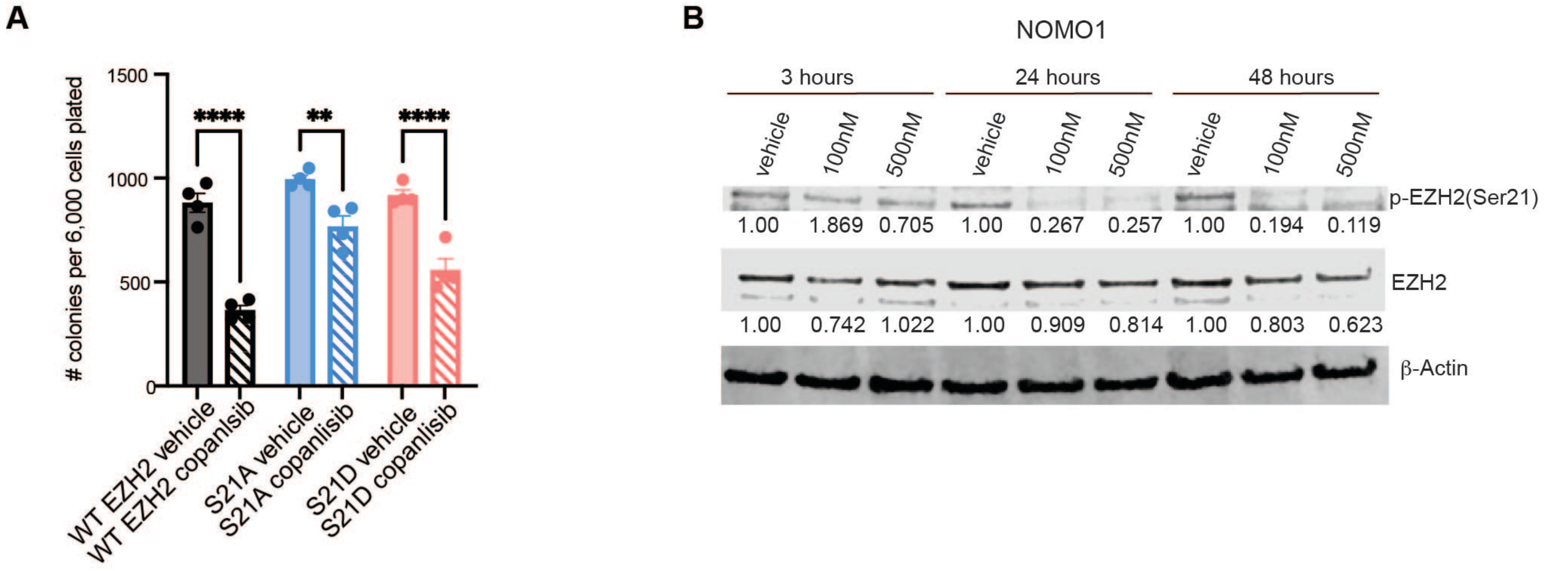

Supplementary Figure 6: PI3K Disruption impairs LSC function via changes in phosphorylation of EZH2 at Ser21 (A) total colony counts in KMT2A-MLLT3 leukemic colonies expressing EZH2ser21 mutants treated with 100nM Copanlisib. **(B)** Western blot analysis showing loss of pEZH2ser21 upon PI3K inhibition in NOMO1 cells treated with 100nM or 500nM Copanlisib for 3, 24, or 48 hours. Each value is presented as mean +/- standard error of the mean (SEM). One-way ANOVA test with Tukey's multiple comparisons was used. ****P ≤ 0.0001 **P ≤ 0.01

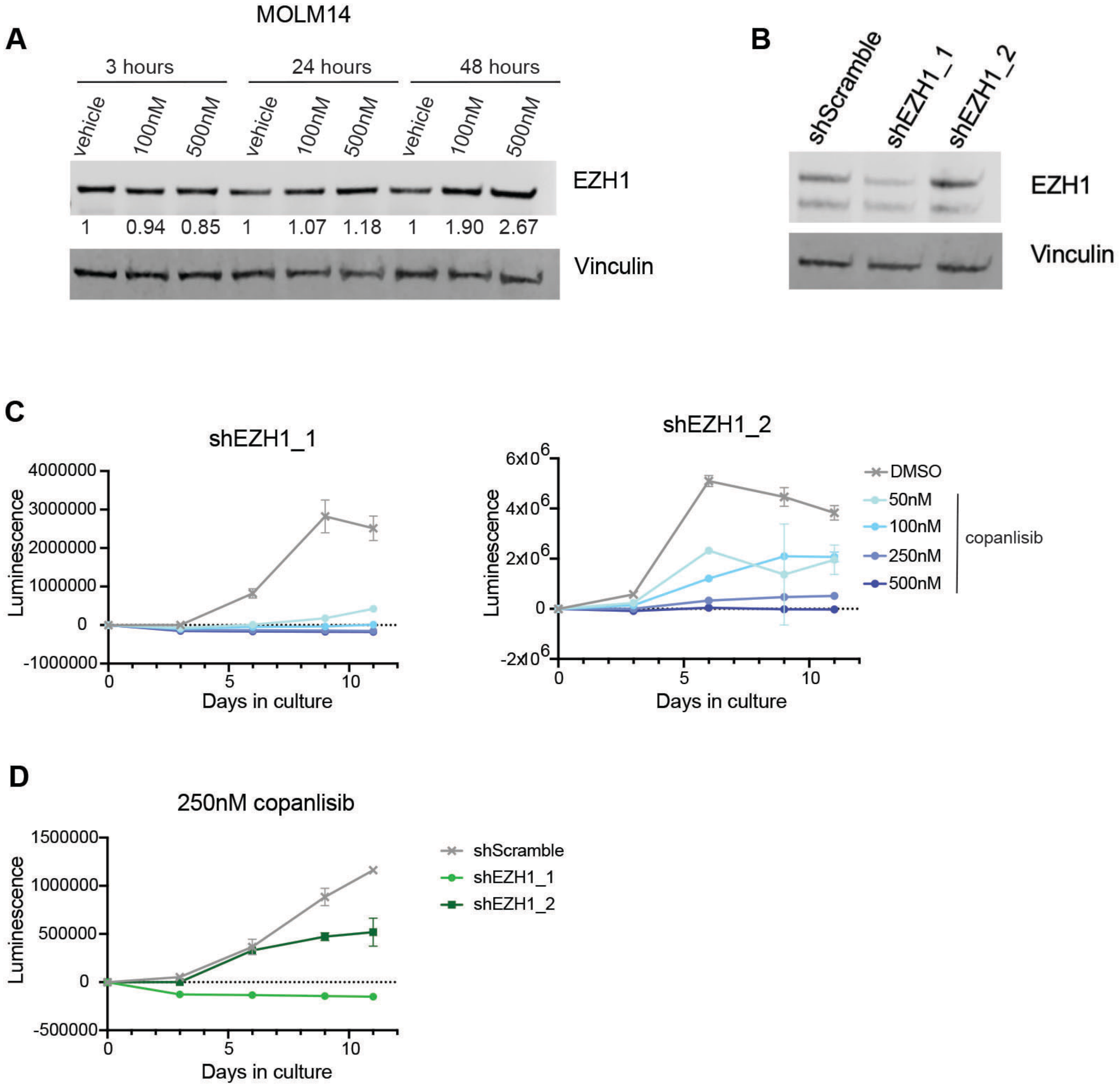

Supplementary Figure 7: EZH1 is Essential After PI3K Inhibition (A) Western blot analysis for EZH1. Quantification shown normalized to vehicle control over vinculin loading control. **(B)** Western blot analysis showing levels of EZH1 knockdown in shEZH1 lines. **(C)** Proliferation assays on NOMO1 cells with shEZH1 knockdown with varying doses of copanlisib. **(D)** Proliferation assay comparing 250nM copanlisib treated NOMO1 cells with shEZH1 knockdown.

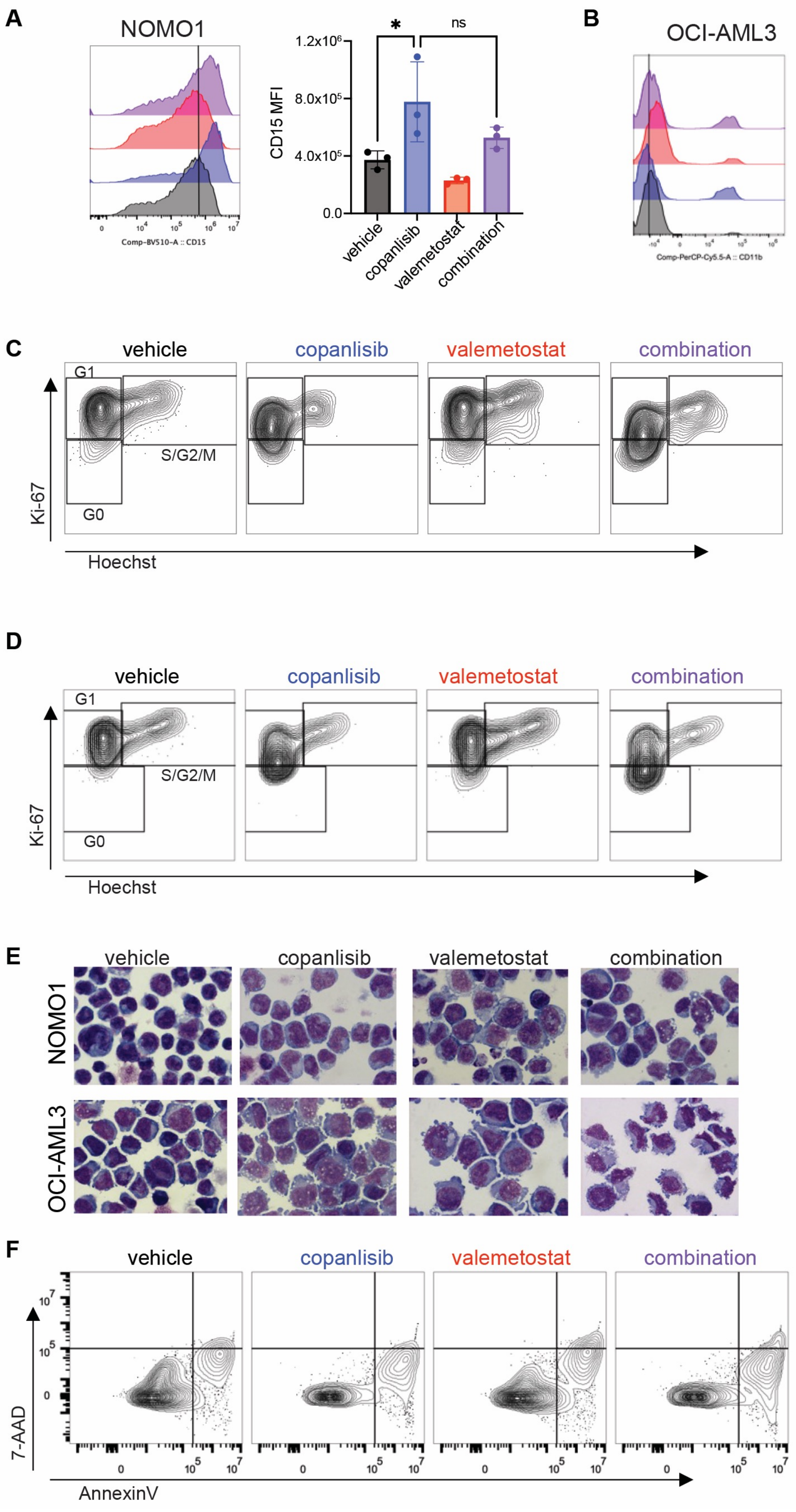

Supplementary Figure 8: PI3K Inhibition Cooperates with EZH1/2 Dual Inhibition (A-B) Representative histograms and quantification of median fluorescence intensity (MFI) of monocytic markers CD115 and CD11b in two different AML cell lines after treatment with either 100nM copanlisib, 500nM valemetostat, or the combination after 6 days of treatment. **(C-D)** Representative flow cytometry plots and quantitative histograms of cell cycle analysis on NOMO1 cells **(C)** or OCI-AML3 cells **(D)** after 2 days of treatment with either 100nM copanlisib, 500nM valemetostat, or the combination. **(E)** Representative images of cytospins made from NOMO1 or OCI-AML3 cells treated for 6 days with either 100nM copanlisib, 500nM valemetostat, or the combination. Captured using 630x objective.**(F)** Representative flow cytometry plots of AnnexinV and 7-AAD staining to measure apoptosis in NOMO1 cells after 6 days of treatment with either 100nM copanlisib, 500nM valemetostat, or the combination. Each value is presented as mean +/- standard error of the mean (SEM). One-way ANOVA test with Tukey's multiple comparisons was used in C-F. ****P $\leq$ 0.0001 **P $\leq$ 0.01 *P$\leq$0.05

A

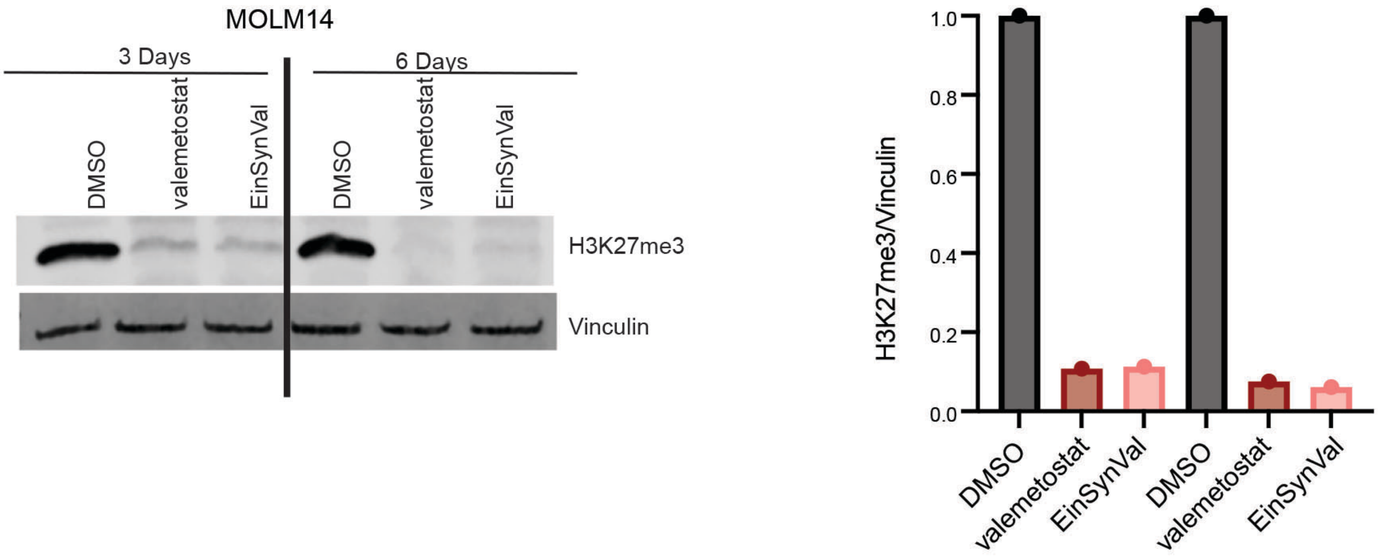

B

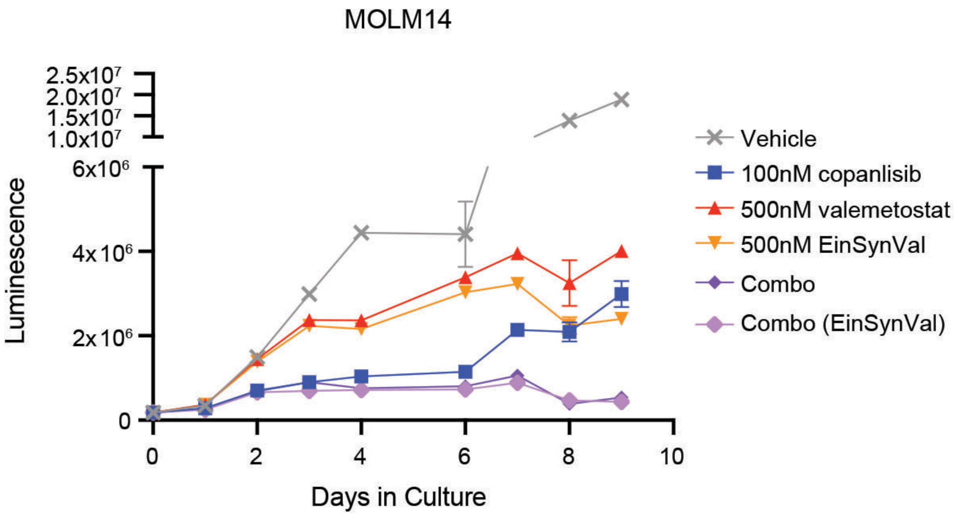

Supplementary Figure 9: Validation of Synthesized EZH1/2 Inhibitor(A) Western blot comparing H3K27me3 levels in MOLM14 cells treated with either Valemetostat (Chemietek) or Einstein Synthesized DS-3201 after 3 and 6 days of treatment. **(B)** Quantification of Western Blot in A normalized to Vinculin loading control. **(C)** CellTiterGlo proliferation assay comparing Valemetostat (chemietek) to Einstein Synthesized DS-3201.

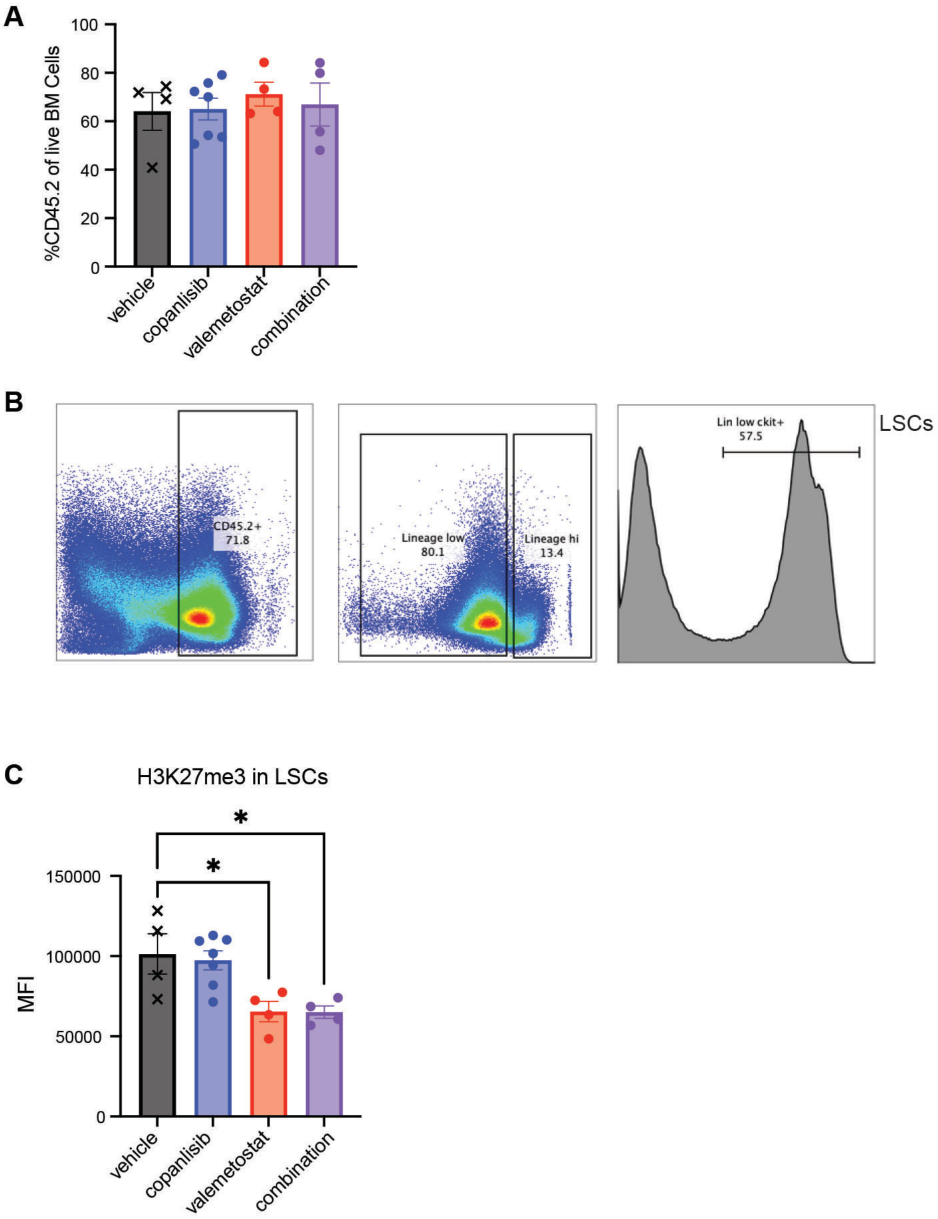

Supplementary Figure 10: EZH1/2 Inhibition Causes Impaired PRC2 Activity in LSCs (A) Flow cytometry analysis of CD45.2 on bone marrow aspirates of NPM1c-NRAS drug treated mice after 2 weeks of treatment. **(B)** Gating strategy for LSCs. **(C)** Quantification of Median Fluorescent Intensity (MFI) of H3K27me3 in LSCs demonstrating on target drug effects in LSCs. Each value is presented as mean +/- standard error of the mean (SEM). One-way ANOVA test with Tukey's multiple comparisons was used in C. *P≤0.05

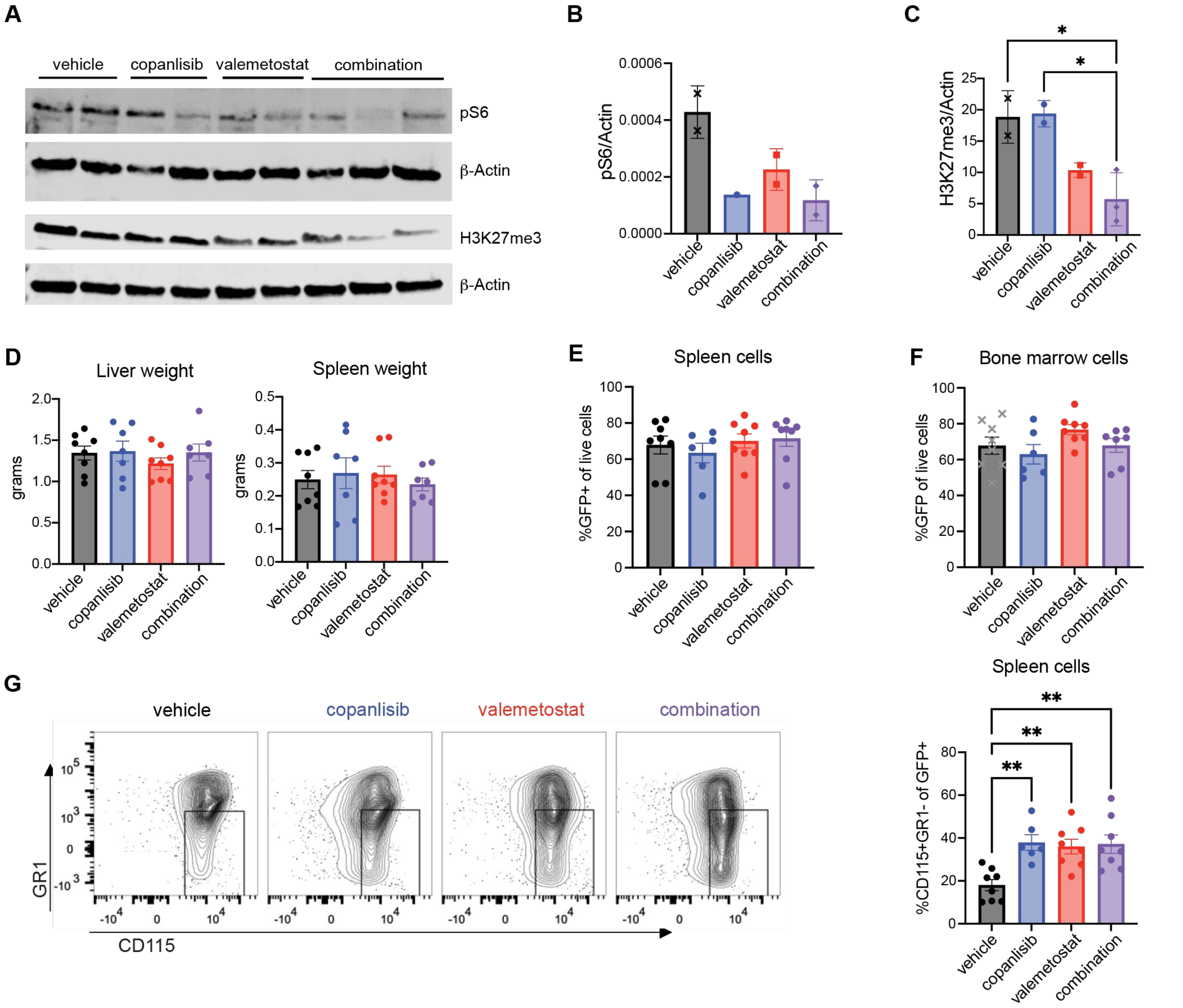

Supplementary Figure 11: PI3K Inhibition Cooperate with EZH1/2 Dual Inhibition to Target LSCs (A-C) Western analysis on spleenocytes from primary transplant recipients (from Fig6A) demonstrating on target drug effects of copanlisib (quantified in **(B)**) and valemetostat (quantified in **(C)**) *in vivo*. One-way ANOVA test with Tukey's multiple comparisons was used to compare signal normalized to β-Actin loading control. $^*P \leq 0.05$ **(D)** Organ weights measured at time of euthanization 2 weeks post treatment. **(E-F)** Disease burden measurement by percent of GFP+ cells among spleenocytes **(E)** and bone marrow cells **(F)** (G) $^{**}P \leq 0.01$, $^*P \leq 0.05$

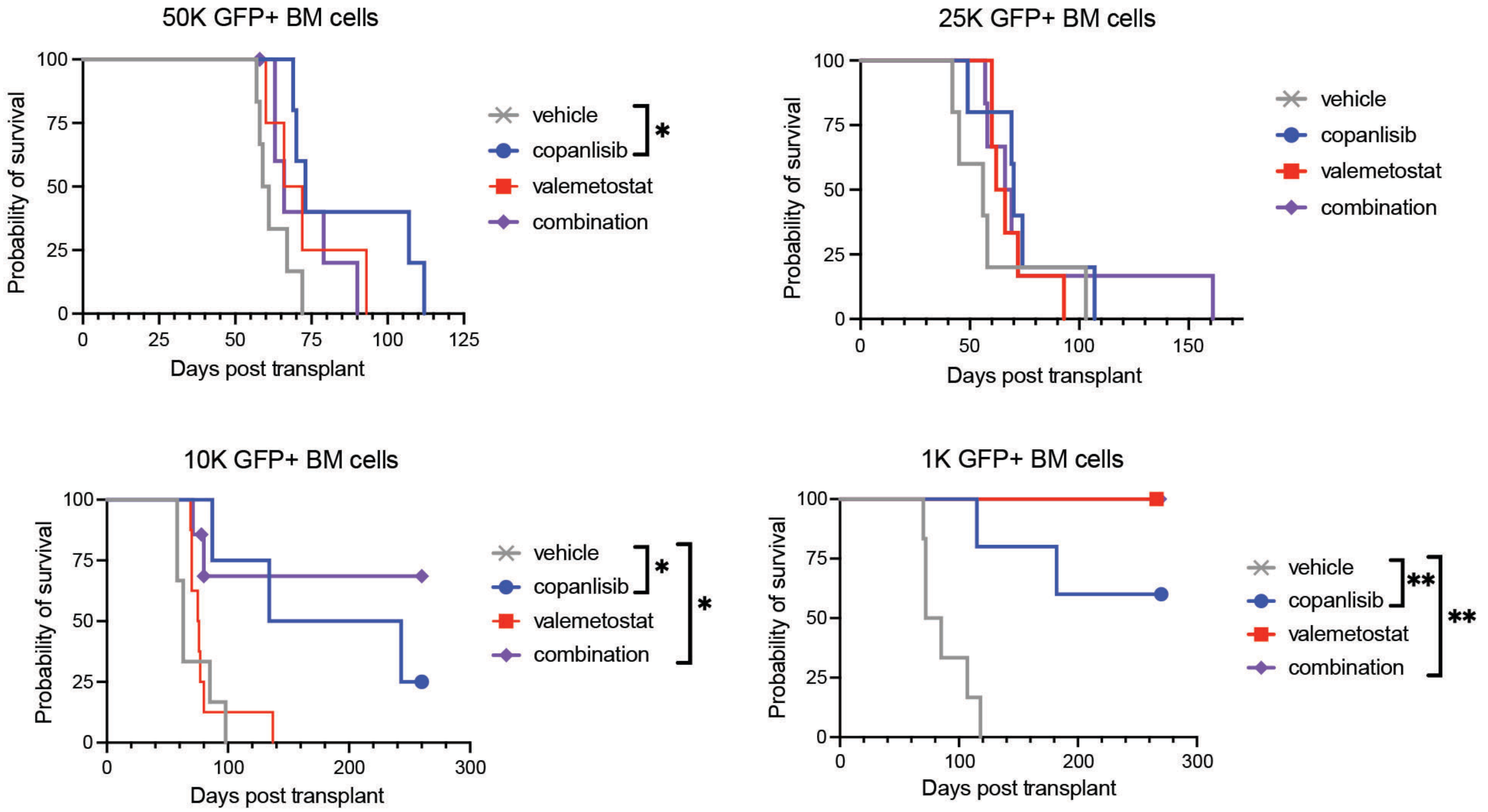

Supplementary Figure 12: PI3K Inhibition Cooperate with EZH1/2 Dual Inhibition to Target LSCs
(Kaplan-Meier survival curves of secondary transplant recipients injected with limiting numbers of KMT2A-MLLT3-GFP+ leukemic cells from primary transplant recipients after copanlisib +/- valemetostat treatment for 2 weeks in primary recipients only. Log-rank analysis was used. $^{**}P \leq 0.01$, $^{*}P \leq 0.05$

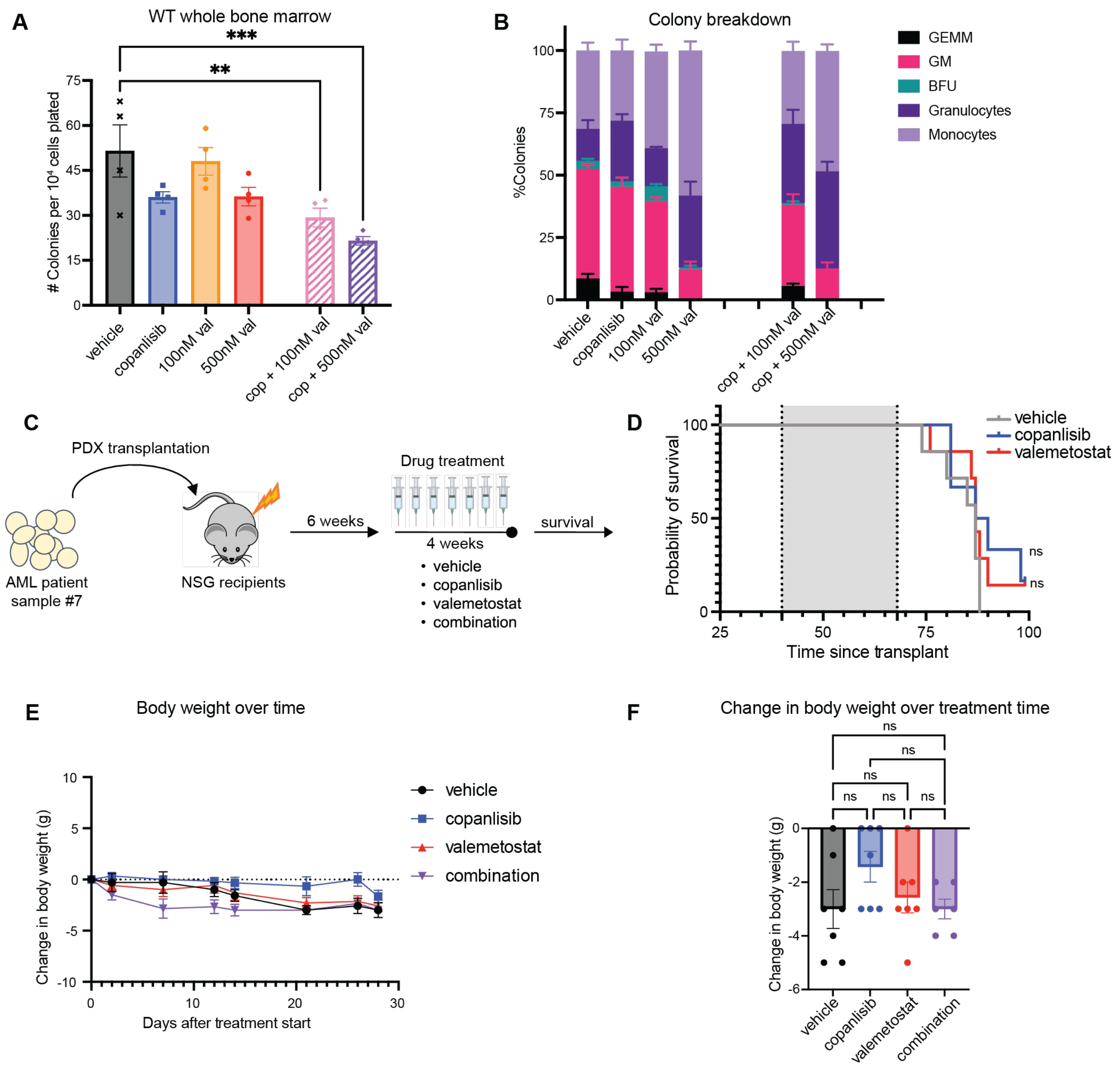

Supplementary Figure 13: There is a reasonable therapeutic window in healthy BM treated with copanlisib and valemetostat (A-B) (A) Colony counts and **(B)** colony breakdown by morphology on colony assays performed on WT mouse BM cells with copanlisib, valemetostat, or the combination of copanlisib + valemetostat (combo) **(C)** Schematic detailing PDX experimental plan. **(D)** Line graph tracking the daily changes of body weight in grams of mice in each treatment group from PDX experiment. **(E)** Bar graph comparing net changes in body weight in grams of last day of treatment compared to first day. Each value is presented as mean +/- standard error of the mean (SEM). One-way ANOVA test with Tukey's multiple comparisons was used. ***P ≤ 0.001 **P ≤ 0.01 *P ≤ 0.05 ns ≥ 0.05.
